# The UAS thioredoxin-like domain of UBXN7 regulates E3 ubiquitin ligase activity of RNF111/Arkadia

**DOI:** 10.1186/s12915-023-01576-4

**Published:** 2023-04-07

**Authors:** Sadek Amhaz, Batiste Boëda, Mouna Chouchène, Sabrina Colasse, Florent Dingli, Damarys Loew, Julien Henri, Céline Prunier, Laurence Levy

**Affiliations:** 1 Sorbonne Université, Inserm, Centre de Recherche Saint-Antoine, CRSA, 75012 Paris, France; 2 Cell Polarity, Migration and Cancer Unit, Institut Pasteur, UMR3691 CNRS, Université Paris Cité, F-75015 Paris, France; 3 CurieCoreTech Mass Spectrometry Proteomics, Institut Curie, PSL Research University, Paris, France; 4 Sorbonne Université, CNRS, IBPS, Laboratoire de Biologie Computationnelle et Quantitative - UMR 7238, 75005 Paris, France

**Keywords:** RNF111, RNF165, TOPORS, UBXN7, RING, Thioredoxin, E3 ubiquitin ligase, SKIL, TGF-β

## Abstract

**Background:**

E3 ubiquitin ligases play critical roles in regulating cellular signaling pathways by inducing ubiquitylation of key components. RNF111/Arkadia is a RING E3 ubiquitin ligase that activates TGF-β signaling by inducing ubiquitylation and proteasomal degradation of the transcriptional repressor SKIL/SnoN. In this study, we have sought to identify novel regulators of the E3 ubiquitin ligase activity of RNF111 by searching for proteins that specifically interacts with its RING domain.

**Results:**

We found that UBXN7, a member of the UBA-UBX family, directly interacts with the RING domain of RNF111 or its related E3 RNF165/ARK2C that shares high sequence homology with RNF111. We showed that UBXN7 docks on RNF111 or RNF165 RING domain through its UAS thioredoxin-like domain. Overexpression of UBXN7 or its UAS domain increases endogenous RNF111, while an UBXN7 mutant devoid of UAS domain has no effect. Conversely, depletion of UBXN7 decreases RNF111 protein level. As a consequence, we found that UBXN7 can modulate degradation of the RNF111 substrate SKIL in response to TGF-β signaling. We further unveiled this mechanism of regulation by showing that docking of the UAS domain of UBXN7 inhibits RNF111 ubiquitylation by preventing interaction of the RING domain with the E2 conjugating enzymes. By analyzing the interactome of the UAS domain of UBXN7, we identified that it also interacts with the RING domain of the E3 TOPORS and similarly regulates its E3 ubiquitin ligase activity by impairing E2 binding.

**Conclusions:**

Taken together, our results demonstrate that UBXN7 acts as a direct regulator for the E3 ubiquitin ligases RNF111, RNF165, and TOPORS and reveal that a thioredoxin-like domain can dock on specific RING domains to regulate their E3 ubiquitin ligase activity.

**Supplementary Information:**

The online version contains supplementary material available at 10.1186/s12915-023-01576-4.

## Background

E3 ubiquitin ligases are key regulators that modulate protein outcome by catalyzing covalent fixation of the ubiquitin protein on a lysine residue of specific substrates. This ubiquitylation happens through a sequential enzymatic reaction involving an E1 activating enzyme that catalyzes covalent binding of ubiquitin to an E2 conjugating enzyme through a thioesther link (E2 ~ Ub), before its transfer to the substrate by the E3 ubiquitin ligase. Multiple rounds of this reaction can lead to poly-ubiquitylation of the substrate since the seven lysine residues (K6, K11, K27, K29, K33, K48, K63), or the N-terminal methionine (M1) of ubiquitin, can be targeted for subsequent attachment of ubiquitin. This chain reaction can generate multiple poly-ubiquitin linkages with distinct functional consequences, the most well-characterized being the proteasome-dependent degradation of substrates modified by K48 poly-ubiquitylation [1]. In some cases, ubiquitylated proteins associated in stable complexes need to be extracted by an unfoldase AAA-ATPase enzyme, VCP/p97, so that they can be degraded by the proteasome or subsequently be assigned to other functions. VCP/p97 is regulated by numerous cofactors including the UBA-UBX family members UBXN7, FAF1, FAF2, SAKS1, and p47/NSFL1C that interact with ubiquitylated proteins via their ubiquitin-associated (UBA) domain and with p97/VCP via their ubiquitin-regulatory X (UBX) domain [2]. UBXN7 also has the ability to interact with neddylated cullins RING E3 ubiquitin Ligase (CRL) via an ubiquitin-interacting motif (UIM) to regulate the turn-over of their substrates such as HIF1α for CRL2^VHL^ [3–6] and NRF2 for CRL3^KEAP1^ [6, 7].

Selectivity of the ubiquitylation reaction relies on E3 ubiquitin ligases specificity for their substrates. E3s can be classified in three main types, depending on the presence of characteristic domains: the homologous to E6-AP C-terminus (HECT), really interesting new gene (RING), and RING-between-RING (RBR) domains [8]. The RING-type ubiquitin ligase family represents the most abundant human E3s with over 600 members [9]. The RING domain has a characteristic “cross-brace” structure of two zinc finger motifs coordinated by eight cysteine/histidine residues and can be classified in two main sub-families depending on the presence and position of the histidines: RING-H2 (C_3_H_2_C_3_) and RING-HC (C_3_HC_4_) [10–12]. In contrast to the HECT and RBR E3s that transfer the ubiquitin from the E2-ubiquitin thioester conjugates (E2 ~ UB) to one of its own catalytic cysteine residues before transferring it to the substrate, RING E3s have no catalytic cysteine and act as a scaffold platform to facilitate direct transfer of the ubiquitin bound to the E2 to a distally recruited substrates. Resolution of the crystal structures of different RING-E2 ~ UB complexes has revealed that E2 ~ UB binding to the RING domain locks E2 ~ UB from an open to a closed conformation that facilitate ubiquitin transfer to the substrate [9, 13, 14].

Many RING E3s are involved in cancer progression and constitutes attractive anti-cancer therapeutic targets [15, 16]. The RING-type E3 ubiquitin ligase RNF111/Arkadia promotes activation of the TGF-β signaling pathway that exerts key roles in cancer progression [17, 18]. TGF-β binding to its receptors TBRI and TBRII leads to SMAD2 and SMAD3 phosphorylation and their subsequent association with SMAD4 as heterotrimeric complexes that activate transcription of specific target genes [17, 18]. Upon TGF-β signal, RNF111 binds phosphorylated SMAD2/3 (P-SMAD2/3) and fosters SMAD-dependent transcription by inducing ubiquitylation and proteasomal degradation of SKIL/SnoN and SKI, two highly related repressors of the TGF-β pathway [19–22]. Beside its important role in TGF-β signaling, RNF111 also has the ability to bind sumoylated proteins and acts as a SUMO-targeted ubiquitin ligase for promyelocytic leukemia (PML) in response to arsenite treatment and for xeroderma pigmentosum C (XPC) in response to UV [23, 24]. RNF111 has also been shown to act as a NEDD8-ligase for histone H4 and cyclic GMP-AMP synthase (cGAS) respectively during DNA damage response induced by ionizing radiation and DNA-triggered innate immune response [25, 26].

RNF111 has a paralog E3 ubiquitin ligase, RNF165/ARK2C/ARKL2, that corresponds to its C-terminal region containing the RING domain [27, 28]. RNF165 is specifically expressed in the nervous system and has been involved in the regulation of BMP signaling [28]. RNF111 and RNF165 RING domains, referred as Arkadia-like RING domains, share high sequence homology and display a similar specific mode of action during the ubiquitin transfer that requires ubiquitin interaction with the RING domain [29, 30]. While the mechanism of action of RNF111 RING domain has been well characterized, only few cellular regulators of RNF111 have been reported so far such as axin [31], RB1CC1 [32], and FHL2 [33].

In this study, we have identified UBXN7 as a novel modulator of RNF111 that directly interacts with RNF111 and RNF165 RING domains through its thioredoxin-like UAS domain. We demonstrated that UBXN7 binding to RNF111 inhibits RNF111 auto-ubiquitylation leading to RNF111 stabilization and modulation of SKIL degradation in response to TGF-β. We then elucidated this molecular mechanism of regulation by showing that docking of the UAS domain of UBXN7 hinders E2s binding to RNF111 RING domain. Through an interactomic study of the UAS domain, we further demonstrated that TOPORS is another E3 ubiquitin ligase that is also directly regulated by UBXN7 via its UAS domain.

## Results

### UBXN7 interacts with RNF111 and RNF165 Arkadia-like RING domains

**Fig. 1:**
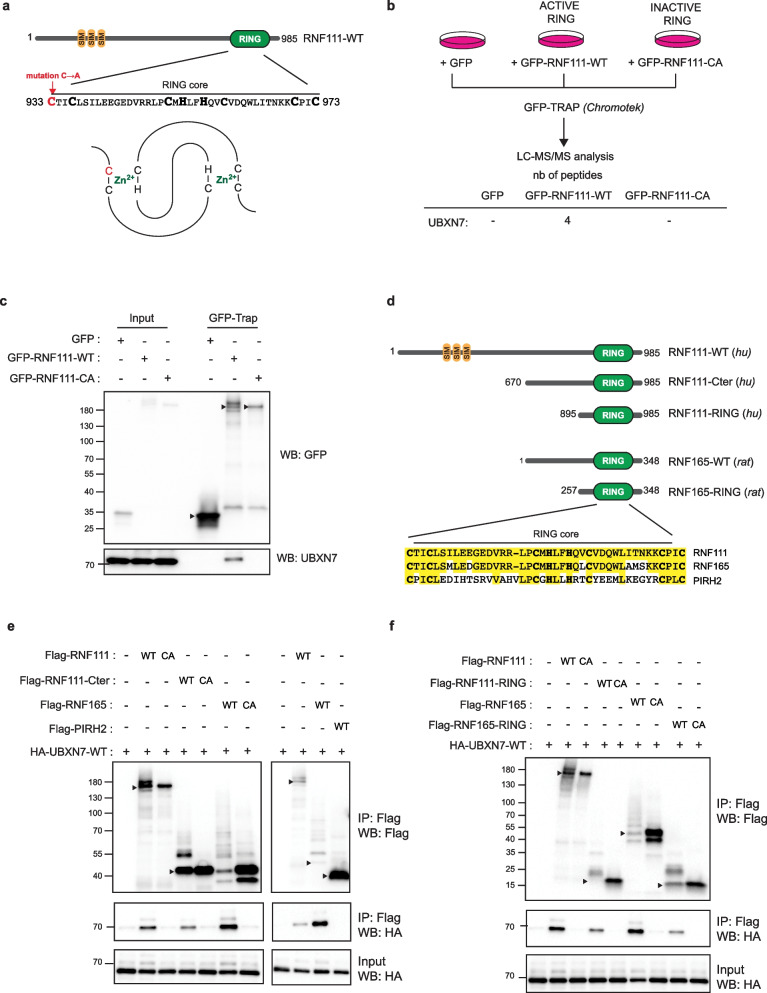
UBXN7 interacts with RNF111 and RNF165 Arkadia-like RING domains. **a** Schematic representation of RNF111 showing its characterized domains: the three SUMO-interacting motifs (SIM) and C-terminal RING domain. The sequence of the RING core is indicated below with the cysteines and histidines of the RING-H2 domain in bold. Mutation of the first cysteine of the RING domain is annotated in red. A schematic representation of the “cross-brace” zinc finger structure of the RING domain is shown [12]. **b** Workflow for the identification of RING specific RNF111 partners. HEK-293 cells were transfected with GFP, GFP-RNF111-WT, or GFP-RNF111-CA before GFP-Trap affinity purification and proteomics mass spectrometry analysis. The number of peptides identified for UBXN7 in each condition is shown below. **c** Endogenous UBXN7 interacts with RNF111-WT but not with RNF111-CA. Whole-cell lysates (Input) and GFP-Trapped lysates (GFP-Trap) from HEK-293 cells transfected with GFP, GFP-RNF111-WT, or GFP-RNF111-CA were analyzed by western blotting with anti-GFP and anti-UBXN7 antibodies. The arrows indicate the protein of interest. **d** Schematic representation of Flag-tagged constructs expressing RNF111 (human) or RNF165 (rat) used in this study. The sequence alignment of the RING core is indicated below. Identical amino acids are highlighted in yellow. The RING core is 80% identical between RNF111 and RNF165. Note that the RING core sequence of RNF165 is identical in rat and human. The sequence alignment with human PIRH2, another RING-H2, is also indicated. **e**,** f** UBXN7 specifically interacts with RNF111 and RNF165 RING domains. Lysates from HEK-293 cells transfected with HA-UBXN7-WT and the indicated Flag-tagged RNF111, RNF165, or PIRH2 constructs were immunoprecipitated with Flag antibody and analyzed by western blotting with anti-Flag and anti-HA antibodies. The corresponding whole-cell lysates (Input) were analyzed with anti-HA antibody. The arrows indicate unmodified RNF111 and RNF165 proteins

We hypothesized that RING E3s might be regulated by proteins that interacts with the RING domain and sought to identify such regulators by comparing the interactome of RNF111-WT to a RING inactivated mutant. Such inactivation can be achieved by mutation of the first cysteine of the RING domain to an alanine (CA) that unfolds the first zinc-finger structure (Fig. 1a), and we have shown previously that this mutation abolishes RNF111 E3 ubiquitin ligase activity, SKIL ubiquitylation, and SMAD-dependent transcription [19, 22]. We then compared the interactome of RNF111-WT and RNF111-CA mutant by performing GFP-Trap affinity purification coupled mass spectrometry on HEK-293 cells individually transfected with GFP-RNF111-WT, GFP-RNF111-CA, and GFP as a negative control (Fig. 1b). Qualitative peptide comparison of the GFP-Trap interactomes enabled detection of RNF111 in the GFP-RNF111-WT and CA conditions, while none in the GFP condition, and allowed us to identify UBXN7 as a potential RING-dependent partner of RNF111 with four peptides detected only in the GFP-RNF111-WT condition. We next confirmed by western blotting that endogenous UBXN7 binds GFP-RNF111-WT but not GFP-RNF111-CA or GFP immobilized on GFP-Trap resins (Fig. 1c) and concluded that UBXN7 might constitute a RING-specific binding partner of RNF111.

The C-terminal region of RNF111 shares approximately 45% sequence homology with its related E3 RNF165, and this sequence homology is particularly strong (> 80%) within the core RING domain [27, 28] (Fig. 1d). We therefore performed Flag co-immunoprecipitation (co-IP) of HA-UBXN7 with Flag-RNF165-WT or the size equivalent C-terminal region of RNF111 (Flag-RNF111-Cter) and found that UBXN7 also interacts with both the RNF111 C-terminal region and RNF165, whereas this interaction is completely abolished when the RING domain is mutated in the first cysteine (Flag-RNF111-Cter-CA and Flag-RNF165-CA) (Fig. 1e, left panel). Importantly, PIRH2, an E3 that equally harbors a RING-H2 type RING domain (Fig. 1d) did not co-immunoprecipitate HA-UBXN7 (Fig. 1e, right panel), indicating that UBXN7 specifically interacts with the Arkadia-like RING domains of RNF111 and RNF165. To circumscribe the interaction region of RNF111, we generated successive deletion inside the C-terminal region of RNF111 and identified that UBXN7 interacts with the region 895 to Cter corresponding to the minimal RING domain as defined in [29] (Additional file 1: Figure S1.a). We then cloned the minimal RING domain of human RNF111 and RNF165 (Flag-RNF111-RING and Flag-RNF165-RING, Additional file 1: Figure S1.b) and performed Flag co-IP of HA-UBXN7-WT with Flag-RNF111-RING and Flag-RNF165-RING to demonstrate that binding of UBXN7 occurs on the minimal RING domain of RNF111 and RNF165 (Fig. 1f).

While RNF111 expected molecular weight is around 107 kDa, RNF111 exhibits an anomalous migratory profile of 170 kDa on acrylamide gel (Fig. 1c, e, f), whereas RNF111-Cter and RNF165 do not (Fig. 1e), this suggests that this migratory anomaly might lie in the N-terminal or central region of RNF111. Noticeably, overexpression of RNF111-WT and RNF165-WT leads to auto-ubiquitylation of RNF111 and RNF165 as shown by the higher molecular weight bands detected with the WT E3s that disappear when the RING domain is mutated (Fig. 1c, e, f). Since UBXN7 contains two ubiquitin-binding domains, UBA and UIM, we then wanted to determine if UBXN7 binds to RNF111 by virtue of its ubiquitylated status. To address this, we performed co-immunoprecipitation of Flag-RNF111-WT or Flag-RNF111-Cter with HA-UBXN7-WT and noticed that the pool of RNF111 bound to HA-UBXN7-WT is not enriched with the higher molecular weight bands corresponding to ubiquitylated RNF111, indicating that RNF111 does not interact with UBXN7 preferentially through its ubiquitylated form (Additional file 1: Figure S1.c).

### The UAS domain of UBXN7 directly interacts with RNF111 RING domain

**Fig. 2:**
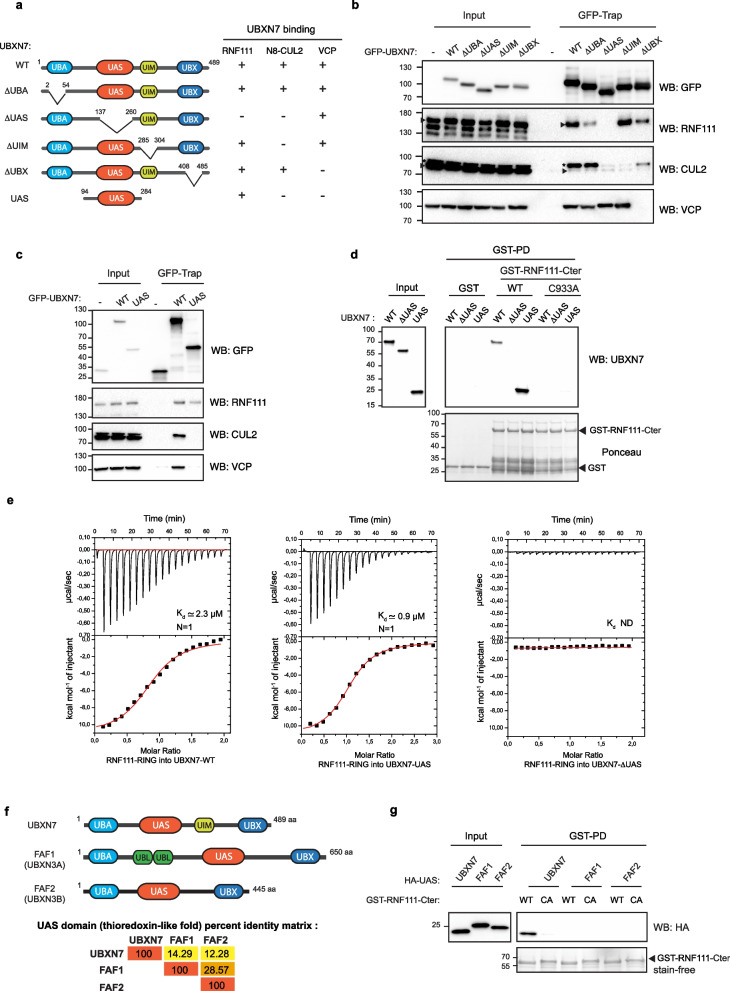
UBXN7 interacts directly with RNF111 RING domain through its UAS domain. **a** Schematic representation of the domain organization of UBXN7 and the different mutants generated, together with their binding capacity to RNF111, N8-CUL2, and VCP observed in **b**. **b**,** c** UBXN7 binds RNF111 via its UAS domain. Whole-cell Lysates (input) and corresponding GFP immunoprecipitated lysates (GFP-Trap) from HEK-293 cells transfected with the different GFP-UBXN7 deletion mutants were analyzed by western blotting with the indicated antibodies. The arrows indicate RNF111 and CUL2. The asterisk indicates neddylated CUL2. **d** The UAS domain of UBXN7 binds directly to RNF111. UBXN7-WT, UBXN7-∆UAS, and UBXN7-UAS recombinant proteins were pulled down with GST, GST-RNF111-Cter-WT, or GST-RNF111-Cter-CA. UBXN7 proteins were revealed by western blotting with anti-UBXN7 antibody. The Ponceau staining shows GST proteins. The input shows the initial amount of each recombinant UBXN7 proteins before pull-down. **e** The RING domain of RNF111 has a binding affinity in the low micromolar range for UBXN7-WT and its UAS domain (*K*_d_ = 2.3 and 0.9 µM, respectively) and a stoichiometry of 1:1 (*N* = 1). ITC experiments were performed by titration of 200 µM of RNF111 RING domain into 10 µM of UBXN7-WT or UBXN7-UAS domain, or into 15 µM of UBXN7-∆UAS at 25 °C. ND not determined. The *K*_d_ values correspond to the average of replicate experiments (UAS, *n* = 3; UBXN7-WT, *n* = 2). ITC data values obtained for each experiments are indicated in Additional file 3: Table S1. **f** Schematic representation of the domain organization of the UAS containing UBA-UBX proteins UBXN7, FAF1, and FAF2, together with the matrix of percent identity of their UAS domain obtained with Clustal Omega multiple sequence alignments. **g** The RNF111 RING domain binds specifically to the UAS domain of UBXN7. HEK-293 lysates transfected with constructs expressing HA-tagged UAS domains from UBXN7, FAF1, or FAF2 were pulled down with GST-RNF111-Cter-WT or GST-RNF111-Cter-CA and subsequently analyzed by western blotting along with the corresponding whole-cell lysates (input). GST proteins were detected with stain-free as a control

To determine precisely which region of UBXN7 interacts with the RING domain of RNF111, we generated UBXN7 deletion mutants for the UBA, UIM, and UBX domains known to interact respectively with ubiquitin, neddylated cullins, and p97/VCP, and for the UAS domain, a thioredoxin-like fold domain that had so far no assigned function (Fig. 2a). We then assessed by GFP-Trap co-IP the ability of endogenous RNF111 to bind these different GFP-Tagged mutants expressed in HEK-293 cells. Deletion of UBA, UBX, and UIM domains had no significant effect on RNF111 binding, while we confirmed that endogenous VCP and neddylated cullin2 (N8-CUL2) binding were completely abolished when respectively UBX and UIM were deleted (Fig. 2b). Unexpectedly, RNF111 binding to UBXN7 was completely abolished when the UAS domains was deleted. VCP binding was retained with the ∆UAS mutant, indicating that deletion of the UAS domain does not affect the whole structure of the protein. However, we noticed that N8-CUL2 binding to UBXN7 was also dependent of the UAS domain. Curiously, involvement of the UAS domain in N8-CUL2 binding was not observed in [3, 5] but was also detected in [4] and might be explained by the close proximity of the UAS and UIM domains. To corroborate the UAS-dependent binding of UBXN7 to RNF111, we confirmed this result in an analogous experiment where HA-tagged version of UBXN7 deletion mutants were expressed and analyzed for binding with GST-RNF111-Cter-WT by GST pull-down (Additional file 2: Figure S2.a). We then cloned the region containing the UAS domain and identified by GFP-Trap experiments that GFP-UBXN7-UAS was sufficient to co-immunoprecipitate endogenous RNF111, but not VCP nor CUL2 (Fig. 2c). Consistently, we demonstrated by in vitro GST pull-down experiments with recombinant proteins that UBXN7-WT and the UAS domain but not UBXN7-∆UAS bind to GST-RNF111-Cter (Fig. 2d), and more precisely that GST-UBXN7-UAS is able to bind the minimal RING domains of both RNF111 (RING-RNF111) and RNF165 (RING-RNF165) (Additional file 2: Figure S2.b). By performing ITC experiments, we identified that UBXN7-WT and the UAS domain of UBXN7 both bind to RING-RNF111 domain with an affinity in the low micromolar range (average *K*_d_ values of 2.3 and 0.9 µM, respectively) and a 1:1 stoichiometry (*N* = 1), while no affinity was detected for UBXN7-∆UAS in the same conditions (Fig. 2e and Additional file 3: Table S1 for individual ITC values). These results demonstrate that the UAS domain of UBXN7 interacts directly with the RING domain of RNF111.

Interestingly, two other members of the UBA-UBX family, FAF1 and FAF2, also exhibit a UAS domain corresponding to a thioredoxin-like fold (Fig. 2f). We then sought to determine if these UAS domains were also able to interact with RNF111. Sequence alignments of the UAS domains of UBXN7, FAF1, and FAF2 (Additional file 4: Figure S3.a) indicate that the UAS domains of FAF1 and FAF2 share 28% identity, whereas the UAS domain of UBXN7 is more divergent, harboring 14% and 12% identity respectively with FAF1 and FAF2 UAS domain (Fig. 2f). Pull-down experiments of HA-tagged UBXN7, FAF1, FAF2 (Additional file 4: Figure S3.b), or their UAS domain (Fig. 2g) with GST-RNF111-Cter indicate that RNF111 does not interact neither with full-length FAF1 and FAF2 nor with their UAS domain. This result is consistent with the sequence divergence of the UAS domain of UBXN7 with FAF1 and FAF2 and highlights that RNF111 specifically interacts with the UAS domain of UBXN7.

### UBXN7 stabilizes RNF111 in a RING and UAS dependent-manner

**Fig. 3:**
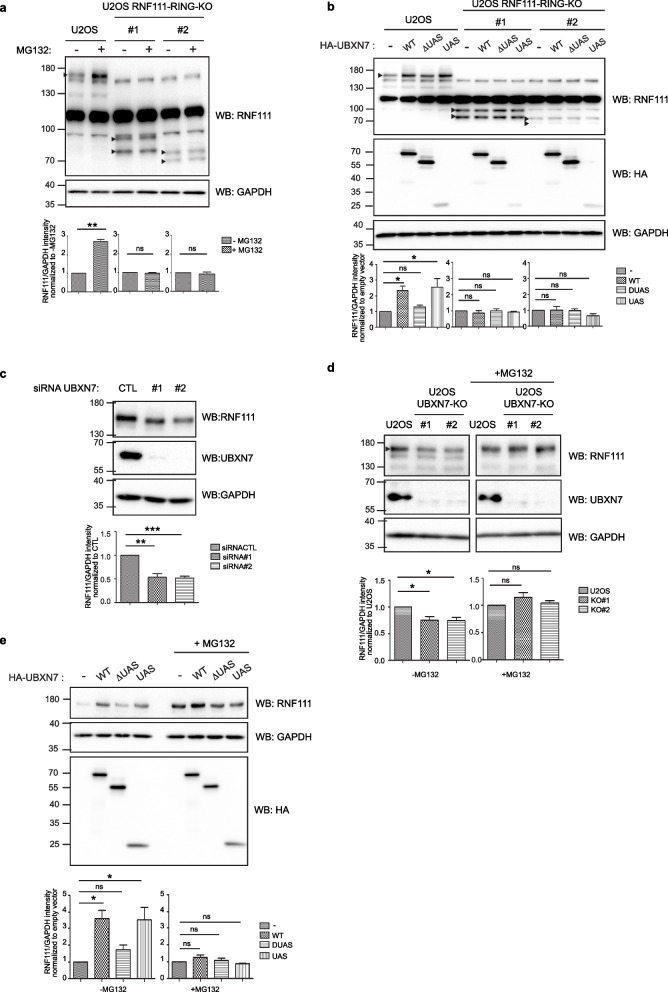
UBXN7 stabilizes RNF111 in a RING and UAS dependent-manner. **a** Endogenous RNF111 is degraded by the proteasome in a RING-dependent manner. Western blotting of lysates from U2OS cells and CRISPR U2OS RNF111-RING-KO clones #1 and #2 treated or not with MG132 for 4 h. Quantification of variations in RNF111 protein level is indicated below the blots panel. Values represent the mean ± SEM of RNF111 intensity normalized to GAPDH loading control intensity and to the indicated control condition from three independent experiments. Statistical analysis was performed using a paired *t*-test. **p < 0.01, ns not significant. Arrows on western blots indicate the band corresponding to RNF111. **b** UBXN7 stabilizes RNF111 in a RING and UAS-dependent manner. Lysates from U2OS cells or U2OS RNF111-RING-KO clones #1 or #2 transiently transfected with HA-tagged empty vector (-), UBXN7-WT (WT), UBXN7-∆UAS (∆UAS), or UBXN7-UAS domain (UAS) were analyzed by western blotting with the indicated antibodies. Quantification of variations in RNF111 protein level was made as in **a** except it was normalized to the empty vector. Statistical analysis was performed using one-way ANOVA with Dunnett’s post test. *p < 0.05, **p < 0.01, ***p < 0.001, ns not significant. **c** Whole-cell extracts of U2OS cells transfected with a control siRNA or 2 independent siRNA #1 and #2 targeting UBXN7 were analyzed by western blotting as indicated. Quantification and statistical analysis as in **b** except that values were normalized to siRNA control. **d** Western blotting analysis of lysates from U2OS cells and U2OS UBXN7-KO clones #1 and #2 treated or not with MG132. Quantification and statistical analysis as in **b** except that values were normalized to U2OS. **e** Western blotting analysis of lysates from U2OS UBXN7-KO clone #1 transfected with the indicated plasmids and treated or not with MG132. Quantification and statistical analysis as in **b**. Western blot quantifications values obtained for each experiments are shown in Additional file 3: Table S1

UBXN7 and RNF111 are both nuclear proteins [5, 23]; it is therefore possible that UBXN7 modulates the E3 ubiquitin ligase activity of RNF111 in the nucleus. We and others have shown previously that RNF111 is an E3 ubiquitin ligase that auto-ubiquitylates [22, 33, 34]. Here we showed that endogenous protein level of RNF111 detected by western-blot on U2OS cell lysates is increased in the presence of the proteasome inhibitor MG132 (Fig. 3a, lanes 1 and 2), suggesting that RNF111 auto-ubiquitylation is associated with RNF111 degradation by the proteasome. To confirm that the RING domain of RN111 is responsible for its proteasomal degradation, we used two independent U2OS RNF111-RING-KO clones (clones #1 and #2) that we engineered previously with the CRISPR technology to express truncated forms of RNF111 corresponding to amino acids 1 to 432 which are therefore devoid of the C-terminal RING domain [22]. Note that western blot analysis confirms that endogenous RNF111 proteins indeed migrate at 170 kDa and not 107 kDa, and that the truncated forms of RNF111 migrate around 80 kDa and not 46 kDa as expected, which confirm that the anomalous RNF111 migration mainly lies in the N-terminal region (Fig. 3a). Interestingly, we showed that proteasomal inhibition does not stabilize the truncated forms of RNF111 in the two U2OS RNF111-RING-KO clones, indicating that the RING domain is responsible for RNF111 degradation by the proteasome. We then set out to determine if UBXN7 interaction with RNF111 RING domain can modulate RNF111 protein level. We found that overexpression of HA-UBXN7-WT or HA-UBXN7-UAS but not HA-UBXN7-∆UAS increases RNF111 protein level whereas this effect is lost in the RNF111-RING-KO clones lacking RNF111 RING domain (Fig. 3b). Conversely, depletion of UBXN7 in U2OS cells transfected with two independent siRNA (#1 and #2) or in two independent CRISPR engineered U2OS clones lacking UBXN7 (U2OS UBXN7-KO clones #1 and #2, Additional file 5: Figure S4) is associated with a decreased level of RNF111 protein (Fig. 3c, d). The decrease of RNF111 protein level in U2OS UBXN7-KO clones is alleviated by inhibition of the proteasome with MG132 treatment (Fig. 3d) or by transient reintroduction of HA-UBXN7-WT or HA-UBXN7-UAS but not HA-UBXN7-∆UAS mutant (Fig. 3e). Consistently, UBXN7 has no effect on RNF111 mRNA level in these different conditions as shown by qRT-PCR analysis (Additional file 6: Figure S5). Altogether, these results indicate that UBXN7 stabilizes RNF111 protein in a RING and UAS-dependent manner.

### UBXN7 inhibits RNF111 auto-ubiquitylation in a UAS-dependent manner

**Fig. 4:**
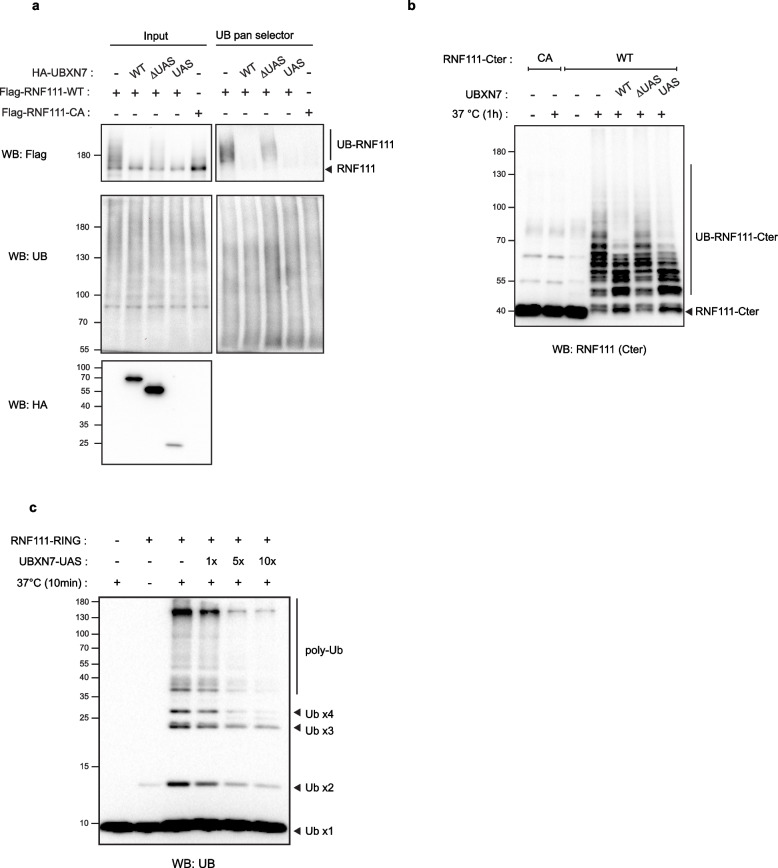
UBXN7 inhibits RNF111 auto-ubiquitylation in a UAS-dependent manner. **a** UBXN7 inhibits RNF111 auto-ubiquitylation in cells. Lysates (input) from U2OS UBXN7-KO clone #1 transiently transfected with Flag-RNF111-WT or Flag-RNF111-CA together with HA-tagged empty vector ( −), UBXN7-WT (WT), UBXN7-∆UAS (∆UAS), or UBXN7-UAS (UAS) were immunoprecipitated with the UB pan selector resin and subsequently analyzed by western blotting. **b** UBXN7 inhibits RNF111 auto-ubiquitylation in vitro. Recombinant RNF111-Cter-CA or WT were incubated at 37 °C for 1 h in the presence of UBE1 (E1), UBE2D2 (E2), ubiquitin (UB), and recombinant UBXN7-WT (WT), UBXN7-∆UAS (∆UAS), or UBXN7-UAS (UAS). RNF111 ubiquitylation was revealed by western blotting using an antibody targeting the RNF111 C-terminal domain. **c** The UAS domain inhibits free ubiquitin chains formation induced by RNF111-RING domain in vitro. Recombinant RNF111-RING was incubated at 37 °C for 10 min in the presence of UBE1 (E1), UBE2D2 (E2), ubiquitin (UB) and 1 to 10 molar excess of recombinant UBXN7-UAS. Ubiquitin chains were revealed by western blotting using an anti-UB antibody

We next explored whether RNF111 stabilization by UBXN7 is due to the inhibition of RNF111 auto-ubiquitylation. We first evaluated ubiquitylation of Flag-RNF111 in U2OS UBXN7-KO cells after transient reintroduction of HA-UBXN7-WT, HA-UBXN7-∆UAS, or HA-UBXN7-UAS, by immunoprecipitating ubiquitylated proteins with a pan UB nanobody resin. As expected, we confirmed that Flag-RNF111-WT is highly ubiquitylated whereas Flag-RNF111-CA is not and found that HA-UBXN7-WT and HA-UBXN7-UAS, but not the HA-UBXN7-∆UAS mutant, prevented RNF111 ubiquitylation (Fig. 4a). To confirm that UBXN7 inhibits auto-ubiquitylation of RNF111, and not ubiquitylation of RNF111 by another E3, we performed in vitro ubiquitylation assays of recombinant RNF111-Cter in the presence of recombinant UBXN7-WT, UBXN7-∆UAS, or UBXN7-UAS proteins. Ubiquitylation of the C-terminal region of RNF111 failed to occur when the RING domain is mutated (RNF111-Cter-CA) and poly-ubiquitylation of RNF111-Cter-WT is dramatically decreased in the presence of UBXN7-WT and UBXN7-UAS, but not UBXN7-∆UAS, which demonstrates that UBXN7 inhibits RNF111 auto-ubiquitylation in a UAS-dependent manner (Fig. 4b). To verify that binding of the UAS domain of UBXN7 inhibits the intrinsic E3 ubiquitin ligase activity of the RING domain, and not only its auto-ubiquitylation, we performed in vitro ubiquitylation assays on the minimal RING domain of RNF111 (RNF111-RING) in the presence of UBXN7-UAS and confirmed that binding of the UAS domain of UBXN7 attenuates RNF111-induced ubiquitin chain formation in a dose dependent manner (Fig. 4c).

### The UAS domain of UBXN7 impedes binding of RNF111 RING domain to E2s

**Fig. 5:**
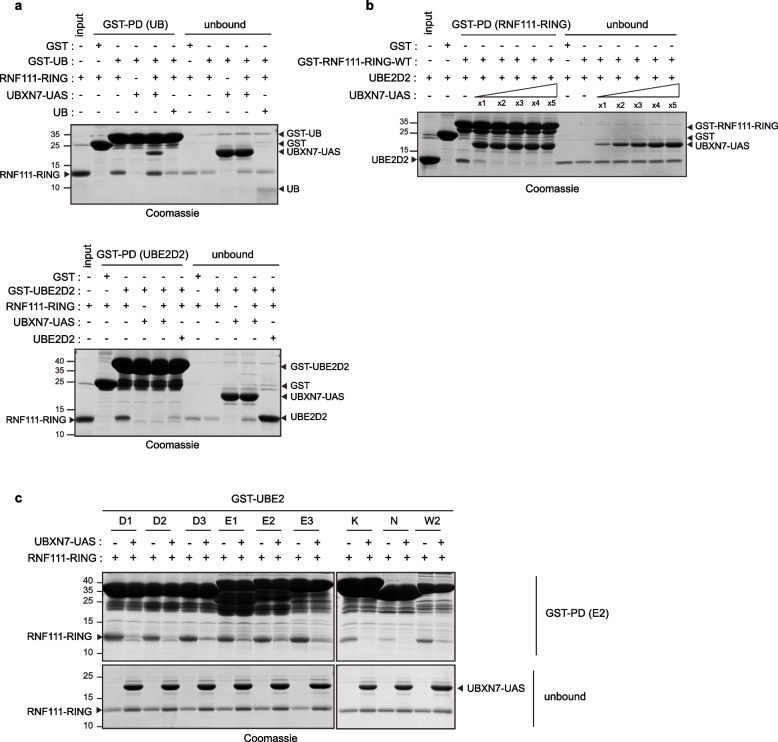
Binding of the UAS domain impedes RING-E2 interaction. **a** The UAS domain competes with RING-E2 but not with RING-UB interaction. GST pull-down (GST-PD) of GST, GST-UB (upper panel) or GST-UBE2D2 (lower panel) with recombinant RNF111 RING, in the presence or not of 10 × molar excess of recombinant UBXN7-UAS. The proteins were revealed on acrylamide gel by Coomassie staining. The unbound panel shows the unbound proteins in the flow-through. Recombinant UB (in the upper panel) and UBE2D2 (in the lower panel) were used as a positive control for binding competition. **b** UBE2D2 binding to GST-RNF111-RING in the presence of increasing amount of UBXN7-UAS. GST pull-down (GST-PD) of GST-RNF111-RING-WT with equimolar concentration of UBE2D2 in the presence of 1 to 5 × molar excess of UBXN7-UAS. The UBE2D2 input and the unbound proteins in the flow-through are shown. **c** The UAS domain inhibits the binding of RNF111-RING with different known interacting E2s. GST pull-down of different GST-E2s (UBE2D1-3, UBE2E1-3, UBE2K, UBE2N, and UBE2W2) with RNF111-RING-WT in the presence or not of 2 × molar excess of UBXN7-UAS. The unbound proteins in the flow-through are shown

We next investigated how interaction of the UAS domain of UBXN7 with the RING domain of RNF111 leads to inhibition of its E3 ubiquitin ligase activity. RING E3s interact with E2 ~ UB to achieve ubiquitin transfer. Crystal structure analyses of RNF165 RING domain in complex with UbcH5b/UBE2D2 ~ UB have highlighted that the Arkadia-like RING domains require interaction of the RING domain with a distal ubiquitin molecule to perform optimal ubiquitin transfer [29, 30]. We therefore went on to determine if binding of UAS to the RING domain of RNF111 could impede its interaction with ubiquitin or E2s. We confirmed by in vitro GST pull-down that RNF111-RING interacts with GST-UB and GST-UBE2D2, whereas, as expected, the UAS domain does not (Fig. 5a). Strikingly, in the presence of both UBXN7-UAS and RNF111-RING, we observed the formation of a tripartite complex between RNF111-RING, UBXN7-UAS, and GST-UB (Fig. 5a upper panel), whereas RNF111-RING binding to GST-UBE2D2 was completely abolished (Fig. 5a lower panel). This demonstrated that UAS binding does not affect the interaction of the RING domain with ubiquitin but dramatically hinders the interaction with the E2 UBE2D2. Moreover, we found that UAS dramatically inhibits UBE2D2 binding to RNF111-RING domain starting from equimolar concentrations (Fig. 5b) and conversely that RING binding to UAS is sustained in the presence of 5 × molar excess of UBE2D2 (Additional file 7: Figure S6). This suggests that RNF111-RING domain has a higher affinity for the UAS domain than for the E2. However, another study has shown that UBE2D2 binding to RNF111-RING domain has a *K*_d_ range of 1 µM [29] equivalent to what we found for UAS (Fig. 2e). One explanation could be that E2 binding to RNF111 RING domain is more dynamic than UAS binding, which would be expected respectively for the binding of an enzyme and an inhibitor. Intriguingly, whereas the UAS domain completely blocks E2 binding at low doses, it only affects significantly RNF111 in vitro ubiquitylation at higher doses as shown in Fig. 4c. Since RNF111 RING domain has the ability to interact with UB, an explanation could be that the E2 charged with UB during the ubiquitylation reaction has an increased affinity compared to the unloaded E2. Finally, all the E2s described to interact with RNF111 that we tested displayed the same binding competition with the UAS domain, demonstrating that the UAS domain constitutes a general inhibitor for all RNF111-E2 complexes formation (Fig. 5c).

### UBXN7 modulates SKIL degradation induced by RNF111 in response to TGF*-*β

**Fig. 6:**
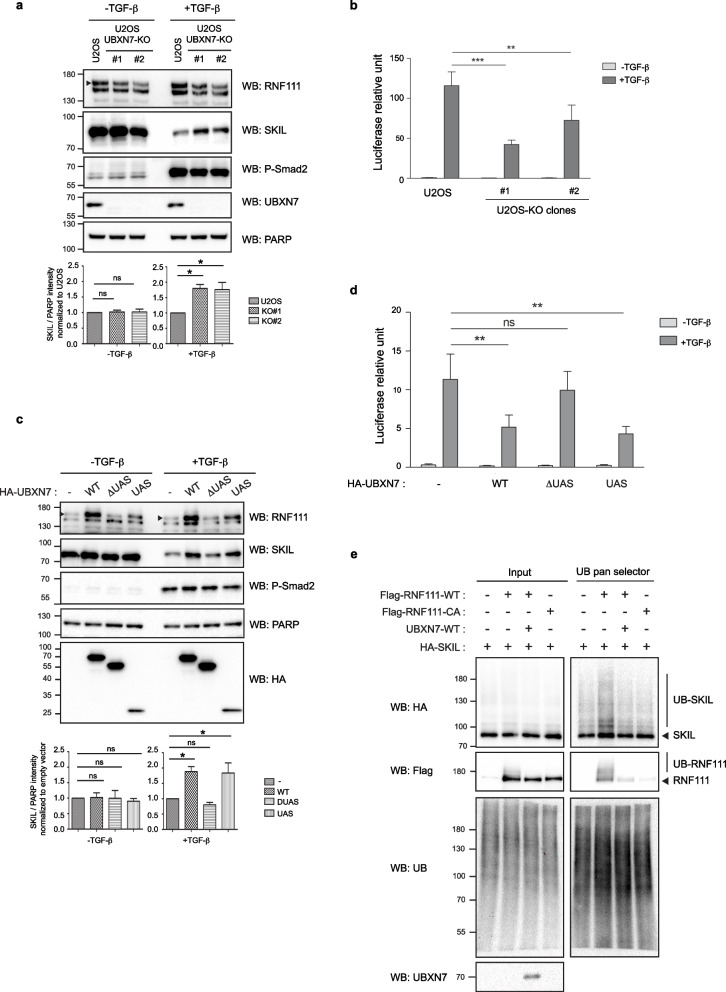
UBXN7 regulates SKIL degradation in a UAS-dependent manner. **a** SKIL increases in the absence of UBXN7. Nuclear extracts from parental U2OS and U2OS UBXN7-KO clones #1 and #2 treated or not with TGF-β for 1 h were analyzed by western blotting. Quantification of SKIL protein level variations is indicated below the blots panel. Values represent the mean ± SEM of SKIL intensity normalized to PARP loading control intensity and to the U2OS control condition in three independent experiments. Statistical analysis was performed using one-way ANOVA with Dunnett’s post test. ns not significant; *p < 0.05. The arrow indicates the band corresponding to RNF111. **b** TGF-β-induced SMAD-dependent transcription is attenuated in the absence of UBXN7. Parental U2OS and U2OS UBXN7-KO clones #1 and #2 were co-transfected with the CAGA_12_-Luc and pRL-TK reporters and treated or not with TGF-β for 8 h before lysis. Data represent means ± SEM of luciferase activities normalized to Renilla in three independent experiments. Statistical analysis was performed using two-way ANOVA with Bonferroni post test on three independent experiments. **p < 0.01, ***p < 0.001. **c** UBXN7 overexpression impairs SKIL degradation in response to TGF-β. Nuclear extracts from U2OS UBXN7-KO clone #1 cells transfected with HA-tagged empty vector (-), UBXN7-WT, UBXN7-∆UAS, or UBXN7-UAS and treated or not for 1 h with TGF-β before extraction were analyzed by western blotting. Quantification and statistics as in **a** except that values were normalized to the empty vector condition. **d** UBXN7 significantly attenuates TGF-β-induced SMAD-dependent transcription in a UAS-dependent manner. U2OS UBXN7-KO clone #1 cells were co-transfected with CAGA_12_-Luc, pRL-TK, and HA-tagged empty vector ( −) or the indicated HA-UBXN7 constructs. Luciferase assays were performed as in **b**. **e** UBXN7 inhibits SKIL ubiquitylation by RNF111. U2OS UBXN7-KO clone #1 cells were transiently transfected as indicated with HA-SKIL, Flag-RNF111-WT, Flag-RNF111-CA, and untagged UBXN7-WT. Lysates (input) were immunoprecipitated with the UB pan selector resin and subsequently analyzed by western blotting. Western blot quantification and luciferase assay values obtained for each experiments are shown in Additional file 3: Table S1

Having shown that UBXN7 regulates RNF111 stability by modulating its E3 ubiquitin ligase activity, we next wanted to determine whether UBXN7 could interfere with RNF111 ability to degrade its substrate SKIL in response to TGF-β signaling. The transcriptional repressor SKIL is degraded after 1 h of TGF-β stimulation and RNF111 E3 ubiquitin ligase activity is essential for this degradation [19–22]. We found that in the absence of UBXN7, the decreased level of RNF111 protein is associated with a less efficient SKIL degradation as shown by comparing SKIL protein level in U2OS parental to U2OS UBXN7-KO clones after 1 h TGF-β stimulation (Fig. 6a). This is associated with an attenuated TGF-β induction of the SMAD-dependent luciferase reporter CAGA_12_-Luc in U2OS UBXN7-KO clones compared to U2OS parental cells (Fig. 6b). These results indicate that in U2OS cells, endogenous UBXN7 is required for an optimal response to TGF-β stimulation. Conversely, we found that HA-UBXN7 overexpression in U2OS UBXN7-KO clones, while stabilizing inactive RNF111, impaired TGF-β dependent SKIL degradation in a UAS-dependent manner (Fig. 6c) to the same extent as overexpression of Flag-RNF111-CA mutant that have an inactive RING domain (Additional file 8: Figure S7). This is associated with a UAS-dependent inhibitory effect of HA-UBXN7 overexpression on SMAD-dependent transcription in response to TGF-β (Fig. 6d). Moreover, by immunoprecipitating with a pan UB nanobody resin the ubiquitylated proteins in U2OS UBXN7-KO cells transfected with HA-SKIL and Flag-RNF111 in the presence or not of overexpressed untagged UBXN7, we found that UBXN7 inhibits HA-SKIL ubiquitylation mediated by Flag-RNF111-WT, to the same extent as mutation of the RING domain in Flag-RNF111-CA (Fig. 6e). Altogether these results show that overexpression of UBXN7 exerts an overall inhibition of RNF111 ubiquitin ligase activity leading to impaired SKIL ubiquitylation and degradation and subsequent attenuation of the SMAD-dependent transcription in response to TGF-β.

### TOPORS is another RING E3 ubiquitin ligase regulated by UBXN7 in a UAS-dependent manner

**Fig. 7:**
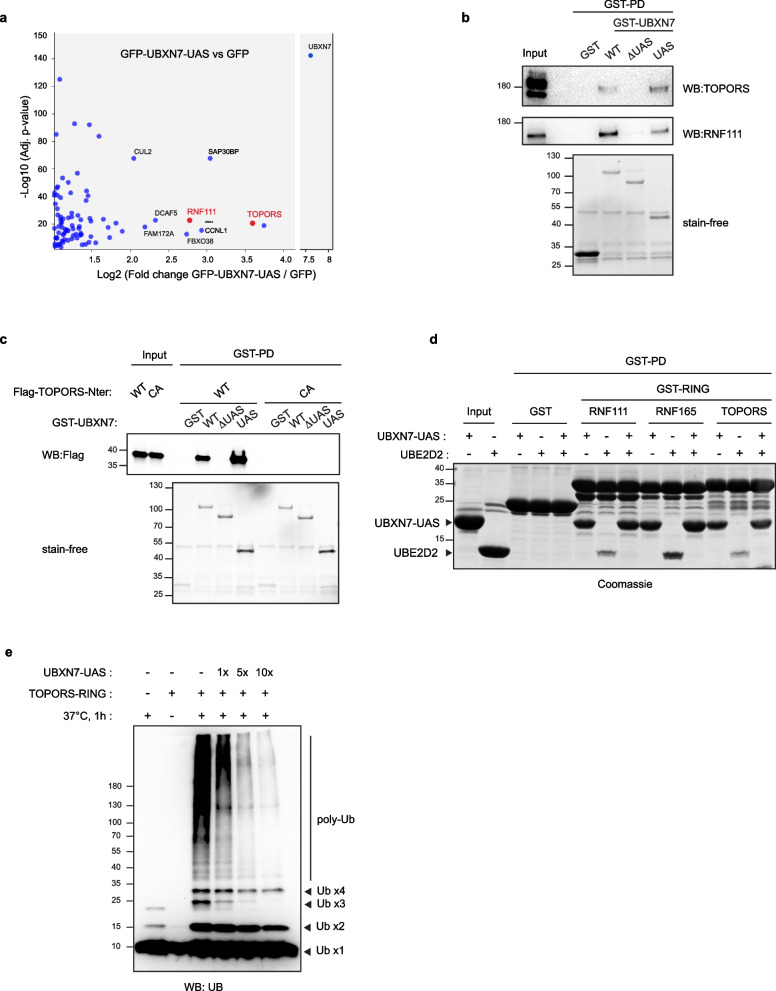
TOPORS is another E3 regulated by the UAS domain of UBXN7. **a** GFP-UBXN7-UAS versus GFP Interactome. Cell lysate from HEK-293 cells transfected with GFP or GFP-UBXN7-UAS were subjected to GFP-Trap affinity purification and co-immunoprecipitated proteins were subsequently identified using quantitative label-free mass spectrometry. Volcano plot illustrating the distribution of the proteins significantly enriched (fold change ≥ 2, *p* value ≤ 0.05, *n* = 5) in GFP-UBXN7-UAS compared to GFP (-Log10 (adj *p* value), *y*-axis; log_2_ fold change GFP-UBXN7-UAS/GFP, *x*-axis). **b**,** c** TOPORS binds to UBXN7-UAS. HEK-293 cellular extracts (**b**) or HEK-293 cellular extracts from cells transfected with Flag-TOPORS-Nter-WT or Flag-TOPORS-Nter-C103A (**c**) were pulled down with the indicated GST-UBXN7 constructs and analyzed by western blotting along with the corresponding cellular extracts (input). The amount of GST proteins in each condition was revealed by stain-free as a control. **d** The UAS domain competes with UBE2D2 for binding to TOPORS RING domain. GST pull-down (GST-PD) of the indicated GST-RING domains with UBE2D2, in the presence or not of UBXN7-UAS. The proteins were revealed on acrylamide gel by Coomassie staining. **e** The UAS domain inhibits free ubiquitin chain formation induced by TOPORS RING domain in vitro. Recombinant TOPORS RING domain was incubated at 37 °C for 1 h in the presence of UBE1 (E1), UBE2D2 (E2), ubiquitin (UB), and 1 × , 5 × , or 10 × molar excess of recombinant UBXN7-UAS (UAS). Free ubiquitin chain formation was revealed by western blotting using an anti-ubiquitin (anti-UB) antibody

We next wanted to determine if the UAS domain of UBXN7 specifically regulates the Arkadia-like E3s or could constitute a more general regulator that interferes with the function of other RING E3s. Indeed, interactomic studies have shown that UBXN7 can interact with many RING containing E3s [3, 35]. To identify exhaustively the proteins that interact with the UAS domain of UBXN7, we performed quantitative GFP-Trap affinity purification coupled to label-free mass spectrometry on HEK-293 cells individually transfected with GFP-UBXN7-UAS and GFP. Quantitative comparison of the GFP-UBXN7-UAS to GFP interactomes identifies 92 significant potential UAS-binding partners (Fig. 7a and Additional file 9: Table S2). Consistently, we found RNF111 among the top 10 most significant hits. Intriguingly, the top 10 hits included several subunits of CRLs such as DCAF5 (CRL4) and FBXO38 (CRL1) and also CUL2 (CRL2) that we could not detect as a UAS interactant by western blotting analysis (Fig. 2c). Nonetheless, this finding corroborates our observation that the UAS domain is involved in UBXN7 interaction with CUL2 (Fig. 2b). Searching for RING domain containing proteins, we identified TOPORS in the top 10 hits as the only additional RING E3 among the 92 significant potential UAS-partners. Note that RNF165 is specifically expressed in neuronal cells and therefore could not be detected in our screen. We confirmed by GST pull-down on cellular extracts with GST-UBXN7 that, similarly to RNF111, UBXN7 interacts with TOPORS in a UAS-dependent manner (Fig. 7b). Furthermore, we showed that the N-terminal region of TOPORS containing the RING domain (Flag-TOPORS-Nter) is sufficient to interact with GST-UBXN7 and that mutation to alanine of the first cysteine of TOPORS RING domain (Flag-TOPORS-Nter-CA) abolishes interaction with UBXN7 as observed for RNF111 (Fig. 7c). As a comparison and to underline RNF111/RNF165 and TOPORS specific interaction with the UAS domain of UBXN7, we confirmed by GST pull-down that the RING containing E3 MUL1 described to interact with and degrade UBXN7 [6, 36] does not interact with UBXN7 via the UAS domain (Additional file 10: Figure S8). Moreover, as observed for RNF111 and RNF165, we found by in vitro GST pull-down experiments that direct binding of the UAS domain of UBXN7 to the minimal RING domain of TOPORS (GST-TOPORS-RING) prevents binding of UBE2D2 (Fig. 7d). This is associated with a remarkable inhibition of the E3 ubiquitin ligase activity of TOPORS RING domain by UBXN7-UAS in in vitro ubiquitylation assays (Fig. 7e), indicating that similarly to RNF111 and RNF165, the UAS domain of UBXN7 inhibits TOPORS E3 ubiquitin ligase activity by interfering with the E2 binding. Curiously, TOPORS and Arkadia-like RING domains do not share strong sequence similarity (Additional file 11: Figure S9.a) suggesting that binding specificity of the UAS domain might be conformational rather than involving a particular motif on the RING domains. Interestingly, we noticed that TOPORS protein level does not increase upon MG132 treatment (Additional file 11: Figure S9.b), suggesting that it is not regulated by auto-ubiquitylation, which is consistent with the fact that we did not observe, as for RNF111, higher molecular weight bands characteristic of auto-ubiquitylation when overexpressing it (Fig. 7c). These results indicate that UBXN7 inhibits TOPORS E3 ubiquitin ligase activity in a similar manner as for RNF111 but does not modulate TOPORS protein level (Additional file 11: Figure S9.c).

## Discussion

In the present work, we have identified UBXN7 as a direct regulator of RNF111. The E3 ubiquitin ligase RNF111 regulates its own protein level by auto-ubiquitylation and we have demonstrated that UBXN7 is involved in this regulation by inhibiting RNF111 auto-ubiquitylation. One of the most well-characterized functions of RNF111 E3 ubiquitin ligase activity is to degrade SKIL in response to TGF-β signaling in order to activate SMAD-dependent transcription [19–22]. We investigated the role of UBXN7 interaction with RNF111 in TGF-β signaling and found that depletion of endogenous UBXN7 decreases the level of RNF111 protein, leading to a less efficient degradation of SKIL and SMAD-dependent transcription in response to TGF-β. On the opposite, overexpression of UBXN7 leads to an increased level of non-functional RNF111 protein associated with an attenuated SKIL degradation and SMAD-dependent transcription in response to TGF-β. These results suggest that endogenous UBXN7, by inhibiting RNF111 auto-ubiquitylation, contributes to maintaining sufficient levels of RNF111 protein necessary for optimal SKIL degradation and TGF-β response, while UBXN7 overexpression completely inhibits RNF111 E3 ubiquitin ligase functions. Our study therefore provides evidence arguing that UBXN7 could act both as an activator or inhibitor of RNF111 functions according to its expression level. It remains to be determined whether an inhibitory action of UBXN7 on RNF111 can be induced by a stimulus that would promote localized interaction of UBXN7 and RNF111. Indeed, beside its role in the TGF-β signaling pathway, RNF111 has been shown to ubiquitylate XPC during the cellular response to UV-induced DNA damage [24], a signaling pathway where UBXN7 has also been involved in association with VCP/p97 [37, 38]. It is therefore possible that regulation of RNF111 ubiquitin ligase activity by UBXN7 might also be required during this process. Moreover, having shown that UBXN7 can also dock on the RNF111-related E3 ubiquitin ligase RNF165, it remains to be determined whether UBXN7 could also regulate the function of this E3 specifically expressed in neurons and involved in motor axon elongation [28].

On the other hand, we did not address in this study the implication of RNF111 on UBXN7 functions. Noteworthy, we could not detect any significant changes in UBXN7 level in U2OS RNF111-RING-KO clones compared to parental U2OS cells, nor identified a UAS-dependent ubiquitylation of UBXN7 by RNF111 (data not shown), suggesting that RNF111 is not involved in UBXN7 stabilization nor ubiquitylation. Interestingly, we showed that the E3 ubiquitin ligase MUL1, known to ubiquitylate and degrade UBXN7, does not interact with UBXN7 through its UAS domain, confirming different mechanisms of regulation of UBXN7 with RNF111 and the E3 involved in its turnover. However, while it has been shown that UBXN7 interacts with neddylated cullins through its UIM domain [3–5], we found that the UAS domain might also be involved in this interaction. Whether RNF111 binding to the UAS domain of UBXN7 could interfere with the UBXN7 function in CRLs regulation needs to be clarified in the future.

Our finding that the UAS domain of UBXN7 acts as a RING docking domain for RNF111 led us to question the specificity of this interaction. Indeed, UBXN7 has been shown to interact with multiple E3 ubiquitin ligases and it was therefore possible that UBXN7 might act as a regulator for different E3s through its UAS domain. To address this question, we have performed a quantitative interactomic study for the UAS domain that revealed a remarkable specificity for RNF111 and TOPORS RING E3s, and further demonstrated that UBXN7 binding has the same inhibitory mechanism on the E3 ubiquitin ligase activity of RNF111, RNF165, and TOPORS RING domains by competing with E2 binding. However, we noted some differences on how the UAS domain of UBXN7 exerts its effect on RNF111 and TOPORS. Noticeably, we provide evidence that, unlike RNF111, TOPORS protein level is not regulated by UBXN7 which is consistent with our observation that TOPORS does not auto-ubiquitylate and is not degraded by the proteasome, suggesting that UBXN7 might only act as an inhibitor for TOPORS function. More intriguingly, while the UAS domain blocks the E2 binding at equimolar concentration both for RNF111 and TOPORS, it seems to have a lower inhibitory effect on RNF111 than TOPORS ubiquitylation in vitro (as shown respectively on Figs. 4c and 7e). Taking into account that RNF111 RING domain has the ability to interact with UB [29, 30], one explanation could be that the E2 charged with UB during the ubiquitylation reaction have an increased affinity compared to the unloaded E2 in the case of RNF111. Noteworthy, since RNF111 and TOPORS RING domains do not share strong sequence homology, intrinsic differences in the interaction of these RING domains with the UAS domain could explain this discrepancy. Crystallography studies will help to understand the specificity of the UAS interaction with RNF111 and TOPORS RING domains and to shed light on their potential differences. TOPORS is an E3 ubiquitin ligase that regulates p53 by ubiquitylation through its N-terminal RING domain and by sumoylation through a distinct C-terminal domain. Whether UBXN7 modulates TOPORS functions needs to be further investigated. Intriguingly, RNF111 and TOPORS are both nuclear proteins involved in sumoylation that localize in PML nuclear bodies, where RNF111 has as a SUMO targeted ubiquitin ligase function on PML [23], and TOPORS acts as a SUMO ligase on IKKε [39]. It will be interesting in the future to determine the entanglement in the regulation by UBXN7 of the SUMO-associated functions of these two E3s.

Noteworthy, our study has assigned a function to the UAS thioredoxin-like domain of UBXN7. While the UAS domain is also found in two other UBA-UBX protein members FAF1 and FAF2, the UAS domain of UBXN7 is more divergent, and we demonstrated that this gives specificity for its docking to RNF111 RING domain. Corroborating this specificity, it has been shown that the UAS domains of FAF1 and FAF2 interacts with long chain unsaturated fatty acids, whereas the UAS domain of UBXN7 does not [40].

Most importantly, our study has unraveled a molecular mechanism of regulation for RNF111 auto-ubiquitylation. It is well established, that most of the E3s are maintained in an off-state to prevent their auto-ubiquitylation or uncontrolled ubiquitylation of their substrates. In most cases, inhibition is achieved by intramolecular interaction on the E3 that masks the accessibility of the E2. This is particularly prevalent among the NEDD4 HECT E3s subfamily that harbor N-terminal C2 and WW domains that can interact with the C-terminal HECT domain [41]. For the 600 putative RING E3s, auto-inhibition by a RING-interacting intramolecular domain have only been described for few E3s, including cIAP1 and CBL with respectively their CARD and LRH domain that interact with the RING domain to inhibit E2–E3 binding [42, 43]. Another example is given by the RBR E3 Parkin, in which the REP domain interacts with the RING1 domain by masking the interaction surface with the E2 [44, 45]. In this study, we have identified that UBXN7, a distinct cellular protein, masks the accessibility of the E2 when bound to the RING domain of RNF111. We found two other examples in the literature of cellular proteins able to directly regulates RING domains, the Glomulin protein that interacts with RBX1 RING domain through a HEAT-like repeat fold [46] and the *Salmonella* protein SopA that interacts with TRIM56 and TRIM65 RING domains through its β-helix and N-lobe domain [47]. Strikingly, as observed for UBXN7, these direct interactions also impede auto-ubiquitylation of the E3s by masking the E2 binding surface of the RING domain [46–49].

## Conclusions

In this study, we have identified that the UAS thioredoxin-like domain of UBXN7, docks on three specific RING domains (RNF111, RNF165 and TOPORS) and demonstrated that it regulates their E3 ubiquitin ligase activity by impairing the E2-RING interaction. Since only few examples of such direct regulators of RING E3s has been reported so far, more investigation in the identification of direct RING regulators will help to determine if this constitutes a more widespread mean to fine-tune RING E3s activity. Whether other thioredoxin-like domains could also be involved in specific RING regulation remains to be discovered.

## Methods

### Plasmid constructions

The following linker DLGGGGSGGGGSGGGGSGSDILNSGGGS was introduced in PEGFP-C1 (Clontech) by annealed oligonucleotides cloning between BglII and EcoRI (PEGFP-C1-linker). The following 3C protease cleavage site LEVLFQGP was introduced in PGEX4T3 (GE healthcare) by annealed oligonucleotide cloning between BamHI and EcoRI (PGEX4T3-3C). pCMV-3xHA-SKIL-WT (HA-SKIL) was described in [22]. Flag-RNF111-WT plasmid corresponding to human RNF111 cDNA isoform 3 (NP_001257458, aa 1–985) cloned in p3xFlag-CMV-10 vector (SIGMA) and Flag-RNF111-CA corresponding to C933A point mutant (substitution to alanine of the first cysteine of the RING-H2 domain) were generated as described in [22] and subcloned in PEGFP-C1-linker (GFP-RNF111-WT and GFP-RNF111-CA). Rat RNF165 cDNA (aa 1–348) was PCR-amplified from MGC cDNA (MRN1768-202722691, Horizon Discovery) and subcloned in p3xFlag-CMV-10 (Flag-RNF165-WT). Flag-RNF165-CA mutant corresponding to C296A (substitution to alanine of the first cysteine of the RING-H2 domain) point mutation was generated by site-directed mutagenesis using quickchange lightning kit (Agilent) and Flag-RNF165-WT as a template. RNF111-Cter (corresponding to human RNF111 aa 670 to Cter), RNF111-RING (aa 895 to Cter), and RNF165-RING (corresponding to rat RNF165 aa 257 to Cter) were generated by PCR amplification using Flag-RNF111-WT or -CA and Flag-RNF165-WT or -CA as a template and subcloned into p3xFlag-CMV-10 (Flag-RNF111-Cter, Flag-RNF111-RING, and Flag-RNF165-RING) and PGEX4T3-3C (GST-RNF111-Cter, GST-RNF111-RING, and GST-RNF165-RING). GST-RNF165-RING was humanized by serine 280 to glycine substitution using mutagenesis. Flag-PIRH2 was generated by PCR amplification of PIRH2 cDNA using GST-PIRH2 (1–261) (Addgene plasmid # 24878, a gift from Cheryl Arrowsmith, [50]) as a template and cloned in p3xFlag-CMV-10. Human cDNA for UBXN7, FAF1, FAF2, TOPORS-Nter (aa 1–247), TOPORS-RING (aa 68–176), and for the different E2s were generated by RT-PCR using iScript cDNA synthesis kit (Bio-Rad) on U2OS or HEK-293 mRNA extracted with Trizol and subsequently cloned into PCMV-3xHA for UBXN7, FAF1, and FAF2 (HA-UBXN7-WT, HA-FAF1, HA-FAF2), into p3xFlag-CMV-10 for TOPORS (Flag-TOPORS-Nter and Flag-TOPORS-RING) and into pGEX4T3-3C for E2s (GST-UBE2D1, GST-UBE2D2, GST-UBE2D3, GST-UBE2E1, GST-UBE2E2, GST-UBE2E3, GST-UBE2K, GST-UBE2N, GST-UBE2W2). Flag-TOPORS-Nter-CA corresponding to C103A point mutant and HA-UBXN7 deletion mutants ∆UBA (∆2–54), ∆UAS (∆137–260), ∆UIM (∆285–304) and ∆UBX (∆408–485) were generated by mutagenesis and Flag-TOPORS-Nter or HA-UBXN7-WT as a template (HA-UBXN7-∆UBA, HA-UBXN7-∆UAS, HA-UBXN7-∆UIM, and HA-UBXN7-∆UBX). The UAS domain of UBXN7 (aa 94–284), FAF1 (aa 316–505), and FAF2 (aa 118–287) were cloned in PCMV-3xHA after PCR amplification using HA-UBXN7, HA-FAF1, and HA-FAF2 as a template (HA-UBXN7-UAS, HA-FAF1-UAS, and HA-FAF2-UAS). All HA-UBXN7 constructs were subcloned into PEGFP-C1-linker (GFP-UBXN7-WT, GFP-UBXN7-∆UBA, GFP-UBXN7-∆UAS, GFP-UBXN7-∆UIM, GFP-UBXN7-∆UBX, and GFP-UBXN7-UAS) and PGEX4T3-3C (GST-UBXN7-WT, GST-UBXN7-∆UBA, GST-UBXN7-∆UAS, GST-UBXN7-∆UIM, GST-UBXN7-∆UBX, and GST-UBXN7-UAS). Expression vector for untagged UBXN7-WT was generated by deletion of the HA tag on PCMV-HA-UBXN7-WT using the quickchange lightning kit (Agilent). SgRNA-UBXN7-ex1 TCTGTGTTGTTGTTCGGCGG targeting exon 1 of the human UBXN7 gene was designed using the CRISPOR software (crispor.org) [51]. Double-stranded oligonucleotides corresponding to SgRNA-UBXN7-ex1 were cloned into pSpCas9(BB)-2A-GFP (a gift from Feng Zhang, Addgene plasmid # 48,140) vector as described in [52]. All constructs were verified by sequencing.

### Cell culture and CRISPR cell line establishment

U2OS human osteosarcoma and HEK-293 human embryonic kidney cells were cultured in DMEM medium containing 10% fetal bovine serum, 100 U/ml penicillin, and 100 µg/ml streptomycin at 37 °C in 5% CO_2_.

For CRISPR cell line establishment, U2OS cells were seeded in six well plates at 80% confluence and transfected with 2 µg of the pSpCas9(BB)-2A-GFP-sgRNA-UBXN7-ex1 constructs using X-tremGENE HP (Sigma) according to the manufacturer’s instructions; 24 h post-transfection, single cells were individually seeded in 96-well plates and cellular clones were amplified. UBXN7 depletion was assessed by western blotting and genomic DNA was extracted from selected UBXN7 depleted clones for PCR amplification of UBXN7 exon1 targeted region with primers AAAGGATGCACTGAGCAGG and AAAGCCCGAAGGAGGAATG. PCR products were subcloned into pCR2.1 vector using TOPO-TA Cloning kit (Invitrogen) and a total of 10 cloned PCR products were sequenced in order to confirm the genomic modification on the different alleles in each CRISPR cellular clones. Two individual clones, UBXN7-KO clones #1 and #2, were selected for subsequent analysis.

### Transfection, cell extracts, immunoprecipitation, and western blotting

Cells were transfected with the appropriate plasmids using X-tremGENE HP (Roche) or the appropriate siRNA (siCTL (D-001810–02), siUBXN7 #1 (J-023533–18) and #2 (J-023533–20), Horizon Discovery) using Dharmafect 3 (Horizon Discovery) according to the manufacturer’s instructions. As indicated, the cells were treated or not for 4 h with MG123 (10 µM) or 1 h with TGF-β (2 ng/ml), and subsequently lysed in the appropriate buffer supplemented with EDTA-free protease Inhibitor (PI, Roche), 10 mM NaF and 10 mM β-glycerophosphate (β). Cell lysates were sonicated (10 s ON, 10 s OFF, 10 s ON) and cleared before quantification with BCA protein assay (Pierce). For whole-cell extract analysis by western blotting, the cells were lysed in RIPA buffer (50 mM Tris–HCl (pH 8), 150 mM NaCl, 1% NP-40, 0.5% sodium deoxycholate, 0.1% sodium dodecyl sulfate). For western blotting analysis on nuclear extracts, the nuclear fraction was isolated by successive extraction with hypotonic buffer (20 mM HEPES, pH 7.5, 10 mM NaCl, 0.2 mM EDTA, 20% glycerol, 1.5 mM MgCl2, 0.1% Triton X-100, 25 mM NaF, 25 mM glycerol phosphate, 1 mM dithiothreitol [DTT]) and hypertonic buffer (hypotonic buffer with 500 mM NaCl) as described in [23].

For GFP immmunoprecipitation (GFP-Trap, ChromoTek Cat# gta-20, RRID:AB_2631357), the cells were lysed in IP150 buffer (150 mM NaCl, 20 mM Tris–HCl (pH7.5), 5 mM EDTA, 1% NP40, 10% glycerol) and 3 mg of proteins were incubated on 25 µl GFP-Trap Chromotek slurry for 1 h and 30 min at 4 °C, followed by 3 washes with IP150 buffer. For Flag-immunoprecipitation, the cells were lysed in IP150 buffer and incubated overnight at 4 °C with 20 µl of Protein G Sepharose 4 FF slurry (Cytiva) and 4 µg of Flag antibody (Sigma) and subsequently washed 3 times with IP150. For immunoprecipitation of ubiquitylated proteins, the cells were treated with 10 µM MG132 for 4 h before lysis in RIPA buffer or extraction of the nuclear fraction; 1 mg of total protein extracts (in RIPA buffer) or nuclear extracts (in hypertonic buffer diluted to 150 mM NaCl with hypotonic buffer) were immunoprecipitated with 20 µl of ubiquitin pan Selector beads slurry (Nanotag Biotechnologies, #N2510) for 1 h at 4 °C followed by 3 washes with RIPA buffer.

Western-blotting was performed using standard procedure. Protein extracts mixed to Laemmli buffer were denaturated at 95 °C for 4 min and subsequently submitted to electrophoresis along with protein ladder standard (PageRuler Prestained protein Ladder, Thermo Scientific) on homemade polyacrylamide gels or precast stain-free gels 4–20% (Biorad). After transfer on nitrocellulose membrane (Biorad), the proteins were revealed using the following antibodies: anti-GFP-HRP (Abcam Cat# ab6663, RRID:AB_305636), anti-Flag-HRP (Sigma-Aldrich Cat# A8592, RRID:AB_439702), anti-HA (Roche Cat# 12,013,819,001, RRID:AB_390917), anti-RNF111 (Abnova Cat# H00054778-M05, RRID:AB_581762), anti-RNF111-Cter (in house), anti-UBXN7 (Novus, NBP2-22,223) or (Thermo Fisher Scientific Cat# PA5-61,972, RRID:AB_2649223), anti-VCP (Santa Cruz Biotechnology Cat# sc-57492, RRID:AB_793927), anti-cullin2 (Thermo Fisher Scientific Cat# 51–1800, RRID:AB_2533898), anti-TOPORS (Novus Cat# NBP1-22,976, RRID:AB_2206015), anti-UB (Santa Cruz Biotechnology Cat# sc-8017, RRID:AB_628423), anti-SKIL (Proteintech Cat# 19,218–1-AP, RRID:AB_10859824), anti-P-SMAD2 (Cell Signaling Technology Cat# 3108, RRID:AB_490941), anti-PARP (Cell Signaling Technology Cat# 9542, RRID:AB_2160739), and anti-GAPDH-HRP (Santa Cruz Biotechnology Cat# sc-47724, RRID:AB_627678). Quantification of protein level variations on western blot was performed by measuring the intensity of the band of interest using ImageLab (BIORAD) normalized to loading control (GAPDH for whole-cell extracts, PARP for nuclear extracts) and to the appropriate control condition in each experiment. Mean of the variations ± standard error of the mean (SEM) were calculated from three independent experiments. Statistical analysis was performed either using a paired *t*-test (for 2 conditions comparison) or a one-way ANOVA with Dunnett’s post test (for more than 2 conditions comparison) on three independent biological experiments.

### Luciferase assay

For luciferase assay, the cells grown in 24-well plates were co-transfected with 0.4 µg CAGA_12_-Luc [53] and 0.1 µg pRL-TK (Promega) or 0.2 µg CAGA_12_-Luc, 0.05 µg pRL-TK, and 0.25 µg HA-UBXN7 constructs; 24 h post-transfection, TGF-β (2 ng/ml) was added for 8 h before lysis in passive lysis buffer (Promega) and successive measurements of luciferase and Renilla activity with the dual-luciferase reporter assay system (Promega) were performed. Luciferase activities were normalized to Renilla activities in technical duplicates. Statistical analyses were performed with two-way ANOVA with Bonferroni post test on three independent experiments using Prism 5.0 software.

### Protein production, purification and GST pull-down

PGEX4T3-3C constructs were transformed in *Escherichia coli* strain BL21 and the proteins were produced at 37 °C in LB media after induction at exponential growth with 0.1 mM isopropyl-β-d-thiogalactopyranoside (IPTG) for 4 h. The proteins were extracted in lysis buffer (50 mM Tris–HCl (pH 8), 150 mM NaCl, 25% glycerol, and protease inhibitors (Roche)) supplemented with 1 mg/ml Lysozyme (Sigma), 0.5% NP40, and 10 U/ml DNAse I (Roche). GST protein concentration in 100 µl of bacterial lysate was quantified on acrylamide gel by comparison against a standard curve of BSA after fixation on 20 µl Glutathione Sepharose 4B slurry (Cytiva). For protein purification, milligrams of the GST-protein of interest contained in cleared bacterial lysate were conjugated to 1 ml of Glutathione Sepharose slurry and incubated overnight with 1 µg of homemade GST-3C protease in 1 ml of 50 mM Tris–HCl (pH 7.5), 150 mM NaCl, and 1 mM DTT. The 1 ml eluate containing the cleaved protein of interest was subsequently incubated for 1 h with 100 µl of Glutathione Sepharose slurry to remove the GST-3C protease. Protein concentration in the eluate was evaluated by quantifying absorbance at 280 nm using a nanodrop spectrophotometer. For GST pull-down, 5 µg of each GST proteins conjugated to 20 µl of Glutathione Sepharose slurry were incubated on wheel for 1 h with cell lysate in buffer IP150 supplemented with PI, NaF and β-glycerophosphate, or with 5 µg of purified recombinant proteins in IP150 supplemented with PI. Beads were washed 3 times with IP150 buffer. When indicated, experiments were performed with IP400 buffer (400 mM NaCl, 20 mM tris pH 7.5, 5 mM EDTA, 1% NP40, 10% glycerol). The proteins were then eluted with Laemmli sample buffer and boiled before loading on acrylamide gel and subsequently detected by gel coloration with Coomassie (InstantBlue, Abcam) or by western blotting. Before immunoblotting, GST proteins were stained as indicated either directly on stain-free precast gel by UV activation or by coloration of the nitrocellulose membrane with Red Ponceau.

### Proteomics mass spectrometry

For GFP-Trap coupled mass spectrometry, affinity purification was performed as above except that the 3 washes were followed by 3 additional washes with buffer ammonium bicarbonate (ABC) 50 mM. Beads were resuspended in 100 µl ABC buffer and the proteins digested by adding 0.2 µg of trypsin-LysC (Promega) for 1 h at 37 °C. Samples were then loaded onto homemade C18 StageTips packed by stacking one AttractSPE® disk (#SPE-Disks-Bio-C18-100.47.20 Affinisep) and 2 mg beads (#186,004,521 SepPak C18 Cartridge Waters) into a 200-µL micropipette tip for desalting. Peptides were eluted using a ratio of 40:60 MeCN:H2O + 0.1% formic acid and vacuum concentrated to dryness. Peptides were reconstituted in injection buffer (2:98 MeCN:H2O + 0.3% TFA) before liquid chromatography tandem mass spectrometry (LC–MS/MS) analysis.

For GFP-RNF111-WT, qualitative interactome experiment was performed with one replicate for each condition (GFP, GFP-RNF111-WT, and GFP-RNF111-CA) by LC–MS/MS using an RSLCnano system (Ultimate 3000, Thermo Fisher Scientific) coupled to an Orbitrap Fusion Tribrid mass spectrometer (Thermo Fisher Scientific). The proteins that displayed no peptides in the GFP and GFP-RNF111-CA conditions and at least 3 distinct peptides identified in the GFP-RNF111-WT condition were selected as potential specific interactants of RNF111 RING domain.

For GFP-UAS, quantitative interactome experiment was performed with five replicates for each condition (GFP and GFP-UAS-UBXN7) by coupling an RSLCnano system to a Q Exactive HF-X (Thermo Scientific). Peptides were first trapped onto a C18 column (75 μm inner diameter × 2 cm; nanoViper Acclaim PepMap™ 100, Thermo Scientific) with buffer A (0.1% formic acid) at a flow rate of 2.5 µL/min over 4 min. Separation was performed on a 50 cm × 75 µm C18 column (nanoViper C18, 3 μm, 100 Å, Acclaim PepMap™ RSLC, Thermo Scientific) regulated to 50 °C and with a linear gradient from 2 to 30% buffet B (100% acetonitrile, 0.1% formic acid) at a flow rate of 300 nL/min over 91 min. MS full scans were performed in the ultrahigh-field Orbitrap mass analyzer in ranges m/z 375–1500 with a resolution of 120,000 at m/z 200. The top 20 intense ions were subjected to Orbitrap for further fragmentation via high energy collision dissociation (HCD) activation and a resolution of 15,000 with the intensity threshold kept at 1.3 × 105. We selected ions with charge state from 2 + to 6 + for screening. Normalized collision energy (NCE) was set at 27 and the dynamic exclusion of 40 s. For identification, the data were searched against the Homo Sapiens UP000005640 database using Sequest HT through proteome discoverer (PD version 2.4). Enzyme specificity was set to trypsin and a maximum of two missed cleavage sites were allowed. Oxidized methionine, N-terminal acetylation, methionine loss, and methionine acetylation loss were set as variable modifications. Maximum allowed mass deviation was set to 10 ppm for monoisotopic precursor ions and 0.02 Da for MS/MS peaks. The resulting files were further processed using myProMS 3.9.3 [54] (https://github.com/bioinfo-pf-curie/myproms). False discovery rate (FDR) was calculated using Percolator [55] and was set to 1% at the peptide level for the whole study. The label-free quantification was performed using peptide extracted ion chromatograms (XICs), reextracted across all conditions and computed with MassChroQ [56], version 2.2.21. XICs from all proteotypic peptides shared between compared conditions (TopN matching) were used and two missed cleavages were allowed. Quantification and statistical analysis of the GFP-UAS-UBXN7/GFP protein ratio based on peptide intensity was performed inside myProMS-Quant v3.6. Median and scale normalization at peptide level was applied on the total signal to correct the XICs for each biological replicate (*N* = 5). To estimate the significance of the change in protein abundance, a linear model (adjusted on peptides and biological replicates) was used and *p*-values were adjusted with the Benjamini–Hochberg FDR procedure. The proteins that display at least 3 corresponding distinct peptides in each five GFP-UAS replicate conditions were considered as UAS interactant under the criteria of a minimum twofold increase between GFP-UAS and the GFP control condition with an adjusted *p *value under 0.05.

### Isothermal titration calorimetry

ITC investigation was done using a VP-ITC calorimeter (Malvern Panalytical) as described in [29]. Purified proteins were dialyzed into a single stock of phosphate-buffered saline, pH 7,4 containing 1 mM Tris (2-carboxyethyl) phosphine hydrochloride (TCEP). All solutions were degassed at room temperature immediately prior to use in the test. Following thermal equilibrium at 25 °C, 10 µl of RNF111 RING domain at 200 µM was injected 20 times every 3.5 min into 10 µM of UBXN7-UAS domain or UBXN7-WT, or into 15 µM of UBXN7-∆UAS. The heat associated with each titration peak was integrated and plotted against the respective molar ratio of titrant and the different ligands. To correct for heats of dilution from the titrant, control experiments were performed by making identical injections of the titrant solution into a cell containing only the buffer and these heat effects were integrated with Origin 7.0 software (OriginLab). Binding stoichiometries and *K*_d_ values were determined by fitting corrected data to a single binding site model.

### Ubiquitylation assays in vitro

In vitro ubiquitylation assay were performed in a 20 µl final volume by mixing 100 nM of purified recombinant RNF111-Cter with 100 nM UBE1, 1 µM UBE2D2, and 1 µM of the indicated purified recombinant UBXN7 proteins (WT, ∆UAS, or UAS), or 2.5 µM of purified recombinant RNF111-RING or TOPORS-RING with 250 nM UBE1, 2.5 µM UBE2D2, and 1 to 10 molar excess of purified recombinant UBXN7-UAS, in the presence of 30 µM ubiquitin, 20 mM Tris–HCl pH 7.5, 50 mM NaCl, 5 mM ATP, 2 mM MgCl2, and 2 mM DTT. Reactions were performed at 37 °C for the indicated time, then stopped by addition of Laemmli buffer and boiling for 5 min, prior to loading on acrylamide gel and revelation by western blotting with anti-RNF111-Cter or anti-ubiquitin antibodies.

## Supplementary Information

**Additional file 1:** **Figure S1. **UBXN7 interacts with RNF111 RING domain. (a) Left panel: Schematic representation of Flag-RNF111 constructs generated by site-directed mutagenesis corresponding to internal successive deletions of the C-terminal region of RNF111 together with the result of their interaction with UBXN7. Right panel: HEK-293 cells were transiently transfected with HA-UBXN7-WT and the indicated Flag-RNF111 constructs, before Flag co-immunoprecipitation ofthe corresponding lysates and western blotting analysis using HA or Flag antibodies. The arrows indicate unmodified RNF111. (b) Sequence alignment (Clustal Omega) of the minimal RING region of human RNF111 and human RNF165 used in this study as defined in [29], along with rat RNF165 RING domain. Note that human and rat RNF165 are highly conserved with only one divergent amino acid in the minimal RING domain (annotated in bold). (c) Lysates from HEK-293 cells transfected with HA-UBXN7-WT and the indicated Flag-tagged RNF111 constructs, were immunoprecipitated with HA antibody and analyzed by western blotting along with the corresponding whole cell lysates (Input). The arrows indicate unmodified RNF111, the asterisk (*) indicates ubiquitylated RNF111.

**Additional file 2:** **Figure S2.**UBXN7-UAS domain interaction with RNF111. (a) Whole cell extracts of HEK-293 cells individually transfected with HA-tagged UBXN7 constructs were pulled down with GST-RNF111-Cter-WT or CA and analyzed by western blotting using anti-HA antibody. The western blot corresponding to the input is shown in the upper panel. The Amount of GST proteins in the samples was revealed by stain-free as a control. (b) The UAS domain of UBXN7 binds directly to RNF111 RING domain. GST pull-down of GST or GST-UBXN7-UAS with recombinant RNF111-RING or RNF165-RING, WT or CA mutants shows direct binding of the RING domains of RNF111 and RNF165 with the UAS domain of UBXN7.

**Additional file 3:** **Table S1. **Data Values. Values for the ITC, western-blot quantification, QRT-PCR and luciferase assays presented in this study.

**Additional file 4:** **Figure S3. **FAF1 and FAF2 interaction with RNF111. (a) Sequence alignment of the UAS core region of human UBXN7, FAF1 and FAF2 corresponding to the thioredoxin-like fold domain using Clustal Omega. (b) HA-UBXN7-WT, HA-FAF1 and HA-FAF2 individually transfected in HEK-293 cells were pulled down with GST-RNF111-Cter-WT or CA and analyzed by western blotting using anti-HA antibody. The western blot corresponding to the input is shown in the upper panel. The Amount of GST proteins in the samples was revealed by stain-free as a control.

**Additional file 5:** **Figure S4. **U2OSUBXN7-KO CRISPR clones #1 and #2 sequences. Genomic UBXN7 exon 1 region is represented with exon 1 in capital letters, start codon (ATG) in bold, sgRNA inred (U2OS parental sequence). Codon translation to UBXN7 protein is indicated below the DNA sequence in blue. Genomic UBXN7 exon 1 region in U2OS UBXN7-KOclones #1 and #2 were amplified by PCR and cloned by TA cloning. For each clone, a total of 10 cloned PCR products were sequenced and the genomic modification detected are shown in red along with the corresponding protein modification in green.

**Additional file 6:** **Figure S5. **UBXN7 does not modulate RNF111 mRNA level. QRT-PCR analysis of RNF111 mRNA level inU2OS, U2OS UBXN7-KO clones #1 and #2 (left panel), and in U2OS UBXN7-KO clone#1 where HA-UBXN7-WT, HA-UBXN7-DUAS and HA-UAS have been transiently reintroduced (right panel). cDNAwere synthetized using the iScript cDNA synthesis kit(Bio-Rad) from 1.5 μg of RNA extracted with Trizol(Invitrogen). QRT-PCR was performed in triplicate using the 2XSYBR Green qPCR master mix(Biotools) according to the manufacturer’s protocol in a Light Cycler 96(Roche). Expression of each gene was calculated by the 2^-ΔΔCt^methods using GAPDH as a control. All data represent mean +/- SEM for three independent experiments. Statistical analyses were performed with One-WayANOVA using Prism 5.0 software. The following primers were used: GAPDH-FTGCACCACCAACTGCTTAGC, GAPDH-R GGCATGGACTGTGGTCATGAG, RNF111-FTTTTGGTGGCGGTGACAGA, RNF111-R ACTCTCCTGTGTCTTTGGTGC. ns: not significant. QRT-PCRvalues are shown in Additional file 3: Table S1.

**Additional file 7:** **Figure S6. **UBXN7-UAS binding to GST-RNF111-RING-WT in presence of increasing amount of E2. GSTpull-down of GST-RNF111-RING-WT with equimolar concentration of UBXN7-UAS inpresence of 1 to 5x molar excess of UBE2D2. The UBXN7-UAS input and the unbound proteins in the flow-through are shown. Proteins were analyzed by Coomassie staining on acrylamide gel.

**Additional file 8:** **Figure S7. **UBXN7 overexpression impairs TGF-b induced SKIL degradation to the same extent as Flag-RNF111-CA overexpression. U2OS UBXN7-KO clone #1 cells were transfected with HA-tagged empty vector, UBXN7-WT, UBXN7-DUAS or UBXN7-UAS, or with Flag-RNF111-CA; 24 h post-transfection, the cells were treated or not for 1 h with TGF-b before extraction of the nuclear fraction. Nuclear extracts were analyzed by western blotting with the indicated antibodies. PARP is used as nuclear loading control. The arrows indicate RNF111 protein.

**Additional file 9:** **Table S2. **GST-UAS interactome hit list. List of the proteins significantly enriched (fold change ≥2, p-value ≤0.05, n=5) in GFP-UBXN7-UAS compared to GFP interactomes.

**Additional file 10:** **Figure S8. **The E3 ubiquitin ligase MUL1 does not interact with UBXN7 through the UAS domain. Flag-MUL1 transfected in HEK-293 cells was pulled down with GST-UBXN7-WT, GST-UBXN7-DUAS or GST-UBXN7-UAS and analyzed by western blotting using anti-Flag antibody. The input and the amount of GST proteins in the samples (stain-free) are shown as acontrol.

**Additional file 11:** **Figure S9. **TOPORS protein level is not stabilized by proteasome inhibition or UBXN7 overexpression. (a) Sequence alignment using Clustal Omega of the RING domain of human RNF111 (isoform 3), RNF165 and TOPORS used in this study. (b) TOPORSis not stabilized upon proteasome inhibition. U2OS cells were treated or not with MG132 for 4h before lysis. Whole cell protein lysates were analyzed by western blotting using the indicated antibodies. GAPDH is used as a loading control. (c) UBXN7 does not stabilize TOPORS. U2OS cells were transfected with HA-tagged empty vector, UBXN7-WT, UBXN7-DUAS or UBXN7-UAS. 24hpost-transfection, cells were treated or not with MG132 for 4h before lysis. Whole cell protein lysates were analyzed by western blotting using the indicated antibodies. GAPDH is used as a loading control.

**Additional file 12.** Figure S10. Images of the original blots presented in this study.

## Data Availability

All data generated or analyzed during this study are included in this published article, its supplementary information files, and publicly available repositories. Supporting data values are included in Additional file 3: Table S1 and images of all the original blots are provided in Additional file 12: Figure S10. The mass spectrometry proteomics data have been deposited to the ProteomeXchange Consortium via the PRIDE [57] partner repository identified with the data set identifier PXD034999.

## Abbreviations

TGF-β: Transforming growth factor-β
RING: Really interesting new gene
UB: Ubiquitin
N8-CUL2: Neddylated-cullin 2
PML: Promyelocytic leukemia
SIM: SUMO-interacting motif
UAS: Thioredoxin-like domain
UBA: Ubiquitin-associated domain
UBX: Ubiquitin-regulatory X domain
UIM: Ubiquitin-interacting motif
VCP: Valosin-containing protein
RBR: RING-between-RING
HECT: Homologous to the E6-AP carboxyl terminus
XPC: Xeroderma pigmentosum C
CRL: Cullin RING ligase
Co-IP: Co-immunoprecipitation
