# Supplementary figures and images for: The UAS thioredoxin-like domain of UBXN7 regulates E3 ubiquitin ligase activity of RNF111/Arkadia
Source: BMC Biol. 2023 Apr 7;21:73. doi: 10.1186/s12915-023-01576-4 (PMC10080908; doi:10.1186/s12915-023-01576-4)

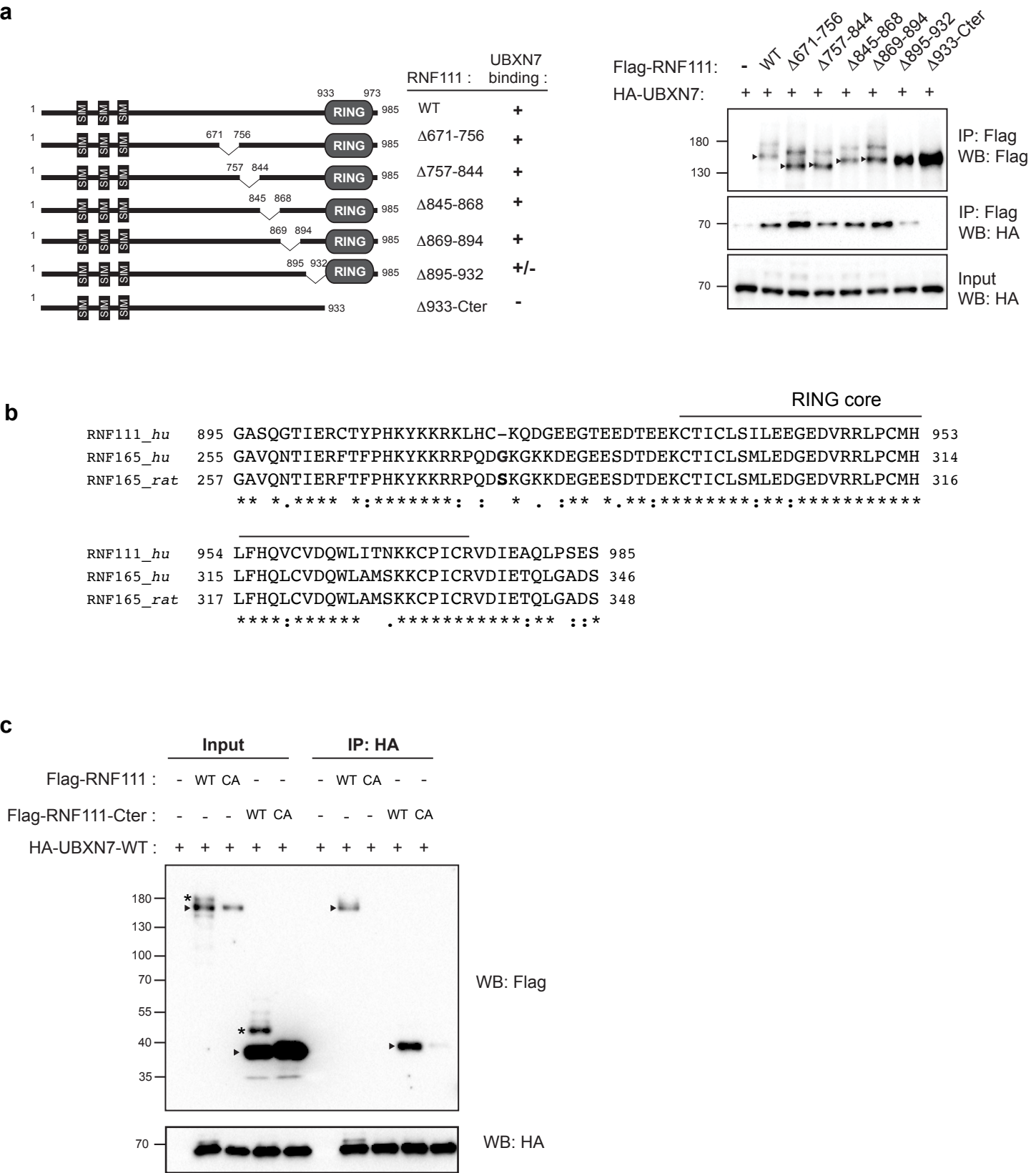

Figure S1

Supplement: Supplementary file 1 — Additional file 1: Figure S1. UBXN7 interacts with RNF111 RING domain. (a) Left panel: Schematic representation of Flag-RNF111 constructs generated by site-directed mutagenesis corresponding to internal successive deletions of the C-terminal region of RNF111 together with the result of their interaction with UBXN7. Right panel: HEK-293 cells were transiently transfected with HA-UBXN7-WT and the indicated Flag-RNF111 constructs, before Flag co-immunoprecipitation ofthe corresponding lysates and western blotting analysis using HA or Flag antibodies. The arrows indicate unmodified RNF111. (b) Sequence alignment (Clustal Omega) of the minimal RING region of human RNF111 and human RNF165 used in this study as defined in [29], along with rat RNF165 RING domain. Note that human and rat RNF165 are highly conserved with only one divergent amino acid in the minimal RING domain (annotated in bold). (c) Lysates from HEK-293 cells transfected with HA-UBXN7-WT and the indicated Flag-tagged RNF111 constructs, were immunoprecipitated with HA antibody and analyzed by western blotting along with the corresponding whole cell lysates (Input). The arrows indicate unmodified RNF111, the asterisk (*) indicates ubiquitylated RNF111. [file 12915_2023_1576_MOESM1_ESM.pdf]

**a**

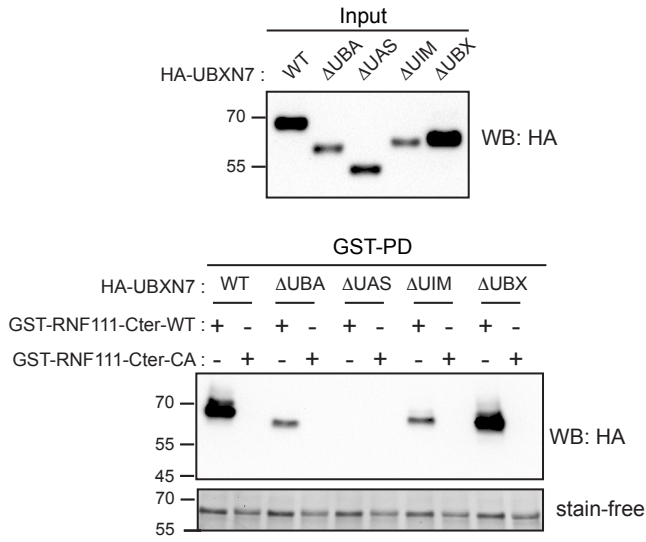

**b**

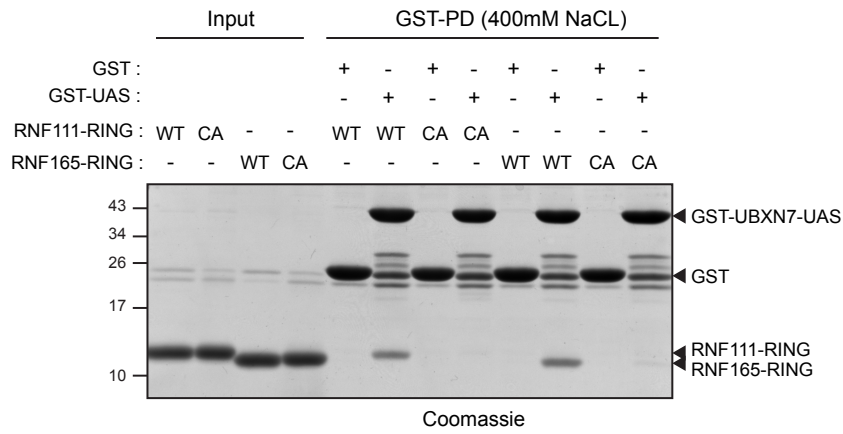

**Figure S2**

Supplement: Supplementary file 2 — Additional file 2: Figure S2.UBXN7-UAS domain interaction with RNF111. (a) Whole cell extracts of HEK-293 cells individually transfected with HA-tagged UBXN7 constructs were pulled down with GST-RNF111-Cter-WT or CA and analyzed by western blotting using anti-HA antibody. The western blot corresponding to the input is shown in the upper panel. The Amount of GST proteins in the samples was revealed by stain-free as a control. (b) The UAS domain of UBXN7 binds directly to RNF111 RING domain. GST pull-down of GST or GST-UBXN7-UAS with recombinant RNF111-RING or RNF165-RING, WT or CA mutants shows direct binding of the RING domains of RNF111 and RNF165 with the UAS domain of UBXN7. [file 12915_2023_1576_MOESM2_ESM.pdf]

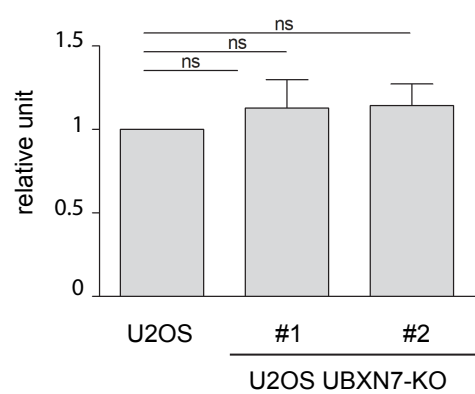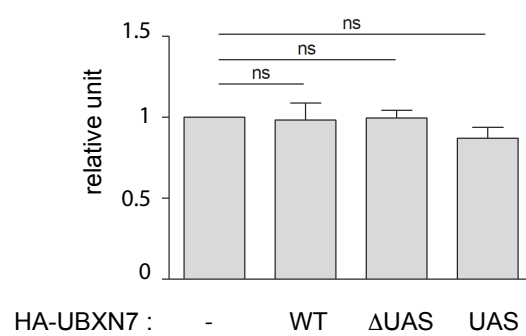

**Figure S5**

Supplement: Supplementary file 6 — Additional file 6: Figure S5. UBXN7 does not modulate RNF111 mRNA level. QRT-PCR analysis of RNF111 mRNA level inU2OS, U2OS UBXN7-KO clones #1 and #2 (left panel), and in U2OS UBXN7-KO clone#1 where HA-UBXN7-WT, HA-UBXN7-DUAS and HA-UAS have been transiently reintroduced (right panel). cDNAwere synthetized using the iScript cDNA synthesis kit(Bio-Rad) from 1.5 μg of RNA extracted with Trizol(Invitrogen). QRT-PCR was performed in triplicate using the 2XSYBR Green qPCR master mix(Biotools) according to the manufacturer’s protocol in a Light Cycler 96(Roche). Expression of each gene was calculated by the 2-ΔΔCtmethods using GAPDH as a control. All data represent mean +/- SEM for three independent experiments. Statistical analyses were performed with One-WayANOVA using Prism 5.0 software. The following primers were used: GAPDH-FTGCACCACCAACTGCTTAGC, GAPDH-R GGCATGGACTGTGGTCATGAG, RNF111-FTTTTGGTGGCGGTGACAGA, RNF111-R ACTCTCCTGTGTCTTTGGTGC. ns: not significant. QRT-PCRvalues are shown in Additional file 3: Table S1. [file 12915_2023_1576_MOESM6_ESM.pdf]

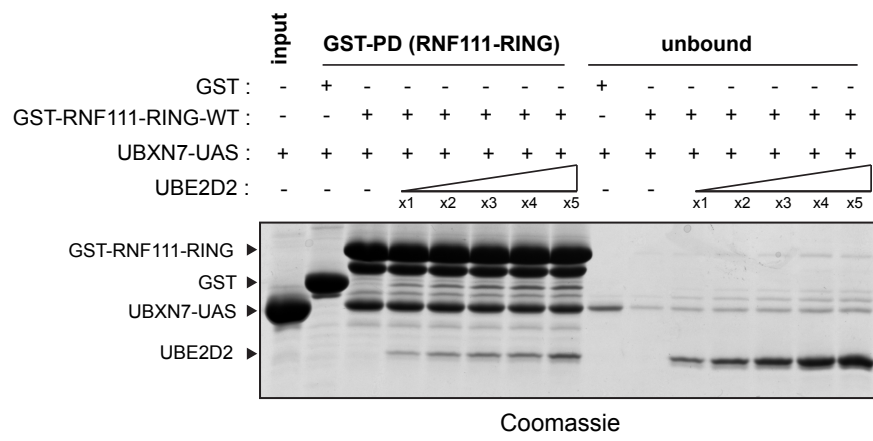

**Figure S6**

Supplement: Supplementary file 7 — Additional file 7: Figure S6. UBXN7-UAS binding to GST-RNF111-RING-WT in presence of increasing amount of E2. GSTpull-down of GST-RNF111-RING-WT with equimolar concentration of UBXN7-UAS inpresence of 1 to 5x molar excess of UBE2D2. The UBXN7-UAS input and the unbound proteins in the flow-through are shown. Proteins were analyzed by Coomassie staining on acrylamide gel. [file 12915_2023_1576_MOESM7_ESM.pdf]

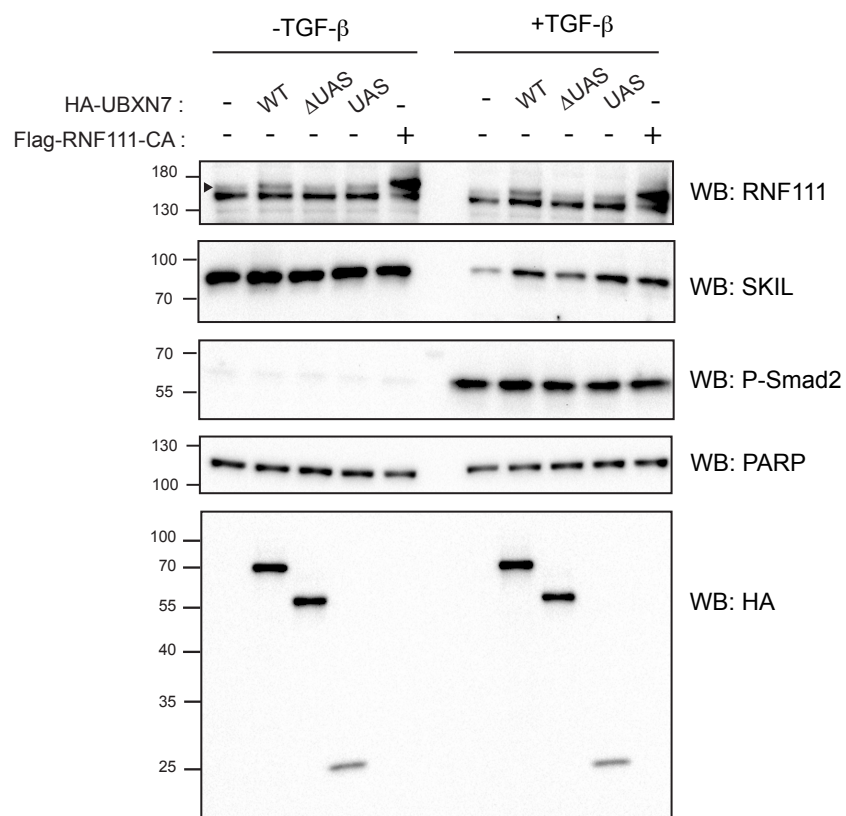

**Figure S7**

Supplement: Supplementary file 8 — Additional file 8: Figure S7. UBXN7 overexpression impairs TGF-b induced SKIL degradation to the same extent as Flag-RNF111-CA overexpression. U2OS UBXN7-KO clone #1 cells were transfected with HA-tagged empty vector, UBXN7-WT, UBXN7-DUAS or UBXN7-UAS, or with Flag-RNF111-CA; 24 h post-transfection, the cells were treated or not for 1 h with TGF-b before extraction of the nuclear fraction. Nuclear extracts were analyzed by western blotting with the indicated antibodies. PARP is used as nuclear loading control. The arrows indicate RNF111 protein. [file 12915_2023_1576_MOESM8_ESM.pdf]

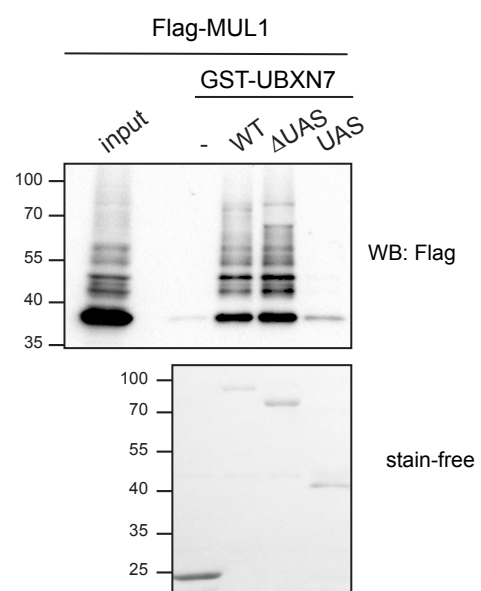

**Figure S8**

Supplement: Supplementary file 10 — Additional file 10: Figure S8. The E3 ubiquitin ligase MUL1 does not interact with UBXN7 through the UAS domain. Flag-MUL1 transfected in HEK-293 cells was pulled down with GST-UBXN7-WT, GST-UBXN7-DUAS or GST-UBXN7-UAS and analyzed by western blotting using anti-Flag antibody. The input and the amount of GST proteins in the samples (stain-free) are shown as acontrol. [file 12915_2023_1576_MOESM10_ESM.pdf]
