# Supplementary material for: The UAS thioredoxin-like domain of UBXN7 regulates E3 ubiquitin ligase activity of RNF111/Arkadia
Source: BMC Biol. 2023 Apr 7;21:73. doi: 10.1186/s12915-023-01576-4 (PMC10080908; doi:10.1186/s12915-023-01576-4)
Supplement: Supplementary file 4 — Additional file 4: Figure S3. FAF1 and FAF2 interaction with RNF111. (a) Sequence alignment of the UAS core region of human UBXN7, FAF1 and FAF2 corresponding to the thioredoxin-like fold domain using Clustal Omega. (b) HA-UBXN7-WT, HA-FAF1 and HA-FAF2 individually transfected in HEK-293 cells were pulled down with GST-RNF111-Cter-WT or CA and analyzed by western blotting using anti-HA antibody. The western blot corresponding to the input is shown in the upper panel. The Amount of GST proteins in the samples was revealed by stain-free as a control. [file 12915_2023_1576_MOESM4_ESM.pdf]

a

```
UBXN7 137    ----TLADLFRPPIDLMHKGSFETAKEC----GQMQNKWLMINIQNVQDFACQCLNRDVW    188
FAF1  321    QFTAEFSSRYGDCHPVFFIGSLEAAFQEAFYVKARDRKLLAIYLHHDESVLTNVFCSQML    395
FAF2  138    SFMHSFEEKYGRAHPVFYQGTYSQALND----AKRELRFLLVYLHGDDHQDSDEFCRNTL    193
                  : . :     ::. *: . * :        : : * : ::  :    : :  :

UBXN7 189    SNEAVKNIIREHFIFWQVYHDSEEGQRY----------------IQFYKLGDFPYVSILD    232
FAF1  396    CAESIVSYLSQNFITWAWDLTKDSNRARFLTMCNRHFGSVVAQTIRTQKTDQFPLFLIIM    455
FAF2  194    CAPEVISLINTRMLFWACSTNKPEGY-------------RVSQAL---RENTYPFLAMIM    237
             .   : . :  .:: *     . ..                   :   : . :* . ::

UBXN7 233    PRTGQK-LVEWH--QLDVSSFLDQVTGFLGE  260
FAF1  456    GKRSSNEVLNVIQGNTTVDELMMRLM-----  481
FAF2  238    LKDRRMTVVGRLEGLIQPDDLINQLT-----  263
              :     ::         ..:: ::
```

b

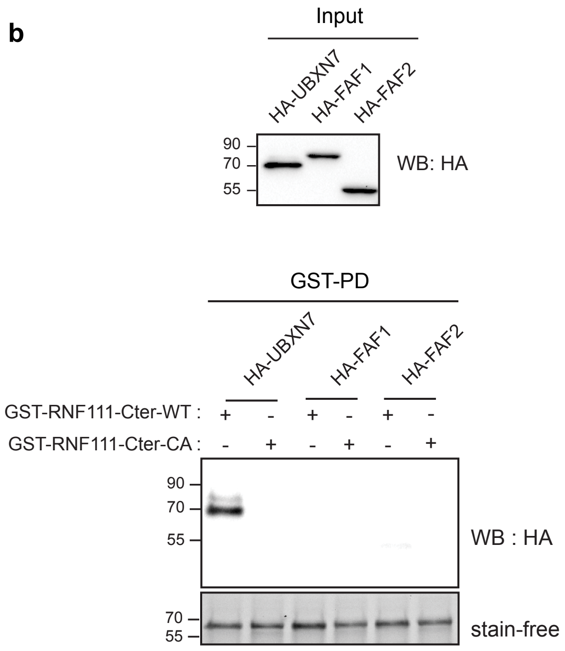

Figure S3
