# Supplementary material for: The UAS thioredoxin-like domain of UBXN7 regulates E3 ubiquitin ligase activity of RNF111/Arkadia
Source: BMC Biol. 2023 Apr 7;21:73. doi: 10.1186/s12915-023-01576-4 (PMC10080908; doi:10.1186/s12915-023-01576-4)
Supplement: Supplementary file 5 — Additional file 5: Figure S4. U2OSUBXN7-KO CRISPR clones #1 and #2 sequences. Genomic UBXN7 exon 1 region is represented with exon 1 in capital letters, start codon (ATG) in bold, sgRNA inred (U2OS parental sequence). Codon translation to UBXN7 protein is indicated below the DNA sequence in blue. Genomic UBXN7 exon 1 region in U2OS UBXN7-KOclones #1 and #2 were amplified by PCR and cloned by TA cloning. For each clone, a total of 10 cloned PCR products were sequenced and the genomic modification detected are shown in red along with the corresponding protein modification in green. [file 12915_2023_1576_MOESM5_ESM.pdf]

• **U2OS parental**

UBXN7 exon 1

```
tctgtgttGTTGTTCGGCGGCGGCGGCGGTAAGATGGCTGCCCACGGGGGCTCCGCGGCGTCCTCGGCG
                                 M  A  A  H  G  G  S  A  A  S  S  A
                             sgRNA
CTGAAGGGGTTAATTCAACAGTTCACCACCATTACCGgtaagagacgc
L  K  G  L  I  Q  Q  F  T  T  I  T  [GASESEV...]
```

• **U2OS UBXN7-KO Clone #1**

```
allele 1  ATGGCTGCCCACGGGGGCTCCGCGGCGTCCTCGGCGCTGAAGGGGTTAATTCAACAGTTCACCACCA-TACCGgtaagagacgc
          M  A  A  H  G  G  S  A  A  S  S  A  L  K  G  L  I  Q  Q  F  T  T  I  P [VQVKV*]

allele 2  ATGGCTGCCCACGGGGGCTCCGCGGCGTCCTCGGCGCTGAAGGGGTTAATTCAACAGTTCACCACCA----CGgtaagagacgc
          M  A  A  H  G  G  S  A  A  S  S  A  L  K  G  L  I  Q  Q  F  T  T  R [CK*]
```

• **U2OS UBXN7-KO Clone #2**

```
allele 1  ATGGCTGCCCACGGGGGCTCCGCGGCGTCCTCGGCGCTGAAGGGGTTAATTCAACAGTTCACCACCAATTACCGgtaagagacgc
          M  A  A  H  G  G  S  A  A  S  S  A  L  K  G  L  I  Q  Q  F  T  T  N [YRCK*]

allele 2  ATGGCTGCCCACGGGGGCTCCGCGGCGTCCTCGGCGCTGAAGGGGTTAATTCAACAGT-----------------aagagacgc
          M  A  A  H  G  G  S  A  A  S  S  A  L  K  G  L  I  Q  Q [*]
```

Figure S4
