# Supplementary material for: The UAS thioredoxin-like domain of UBXN7 regulates E3 ubiquitin ligase activity of RNF111/Arkadia
Source: BMC Biol. 2023 Apr 7;21:73. doi: 10.1186/s12915-023-01576-4 (PMC10080908; doi:10.1186/s12915-023-01576-4)
Supplement: Supplementary file 11 — Additional file 11: Figure S9. TOPORS protein level is not stabilized by proteasome inhibition or UBXN7 overexpression. (a) Sequence alignment using Clustal Omega of the RING domain of human RNF111 (isoform 3), RNF165 and TOPORS used in this study. (b) TOPORSis not stabilized upon proteasome inhibition. U2OS cells were treated or not with MG132 for 4h before lysis. Whole cell protein lysates were analyzed by western blotting using the indicated antibodies. GAPDH is used as a loading control. (c) UBXN7 does not stabilize TOPORS. U2OS cells were transfected with HA-tagged empty vector, UBXN7-WT, UBXN7-DUAS or UBXN7-UAS. 24hpost-transfection, cells were treated or not with MG132 for 4h before lysis. Whole cell protein lysates were analyzed by western blotting using the indicated antibodies. GAPDH is used as a loading control. [file 12915_2023_1576_MOESM11_ESM.pdf]

a

```
TOPORS      68 ------MASAAKEFKMDNFSPKAGTSKLQQTVPADASPDSKCPICLDRFDNVSYL--DRC 119
RNF111     895 GASQGTIERCTYPHKYKKRKLHC---KQDGEEGTEEDTEEKCTICLSILEEGEDVRRLPC 851
RNF165     255 GAVQNTIERFTFPHKYKKRRPQDG--KGKKDEGEESDTDEKCTICLSMLEDGEDVRRLPC 312
                     :   :  .* .:   :    * .     : . :.** ***. ::: . :    *

TOPORS     120 LHKFCFRCVQEWSKNKAECPLCKQPFDSIFHSVRAEDDFKEYVLRPSYNGSFVTPDR    176
RNF111     850 MHLFHQVCVDQWLITNKKCPICRVDIEAQLPSES-----------------------    995
RNF165     313 MHLFHQLCVDQWLAMSKKCPICRVDIETQLGADS-----------------------    346
               :* *   **::*   . :**:*:  ::: : :
```

b

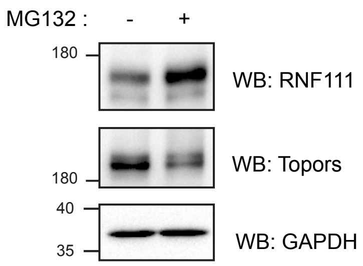

c

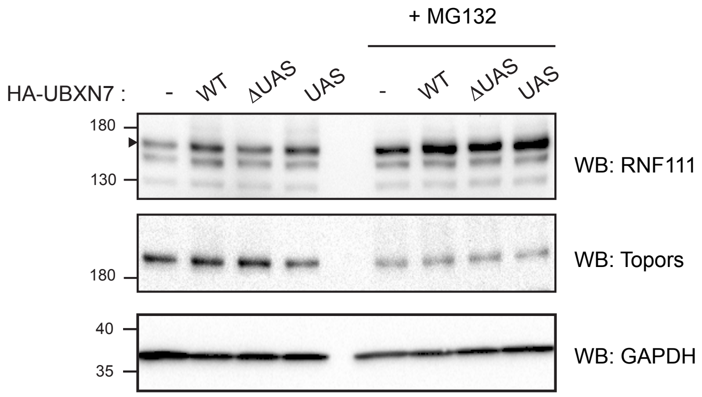

Figure S9
