# Supplementary material for: The UAS thioredoxin-like domain of UBXN7 regulates E3 ubiquitin ligase activity of RNF111/Arkadia
Source: BMC Biol. 2023 Apr 7;21:73. doi: 10.1186/s12915-023-01576-4 (PMC10080908; doi:10.1186/s12915-023-01576-4)

Fig.1.c

WB : GFP

WB : UBXN7

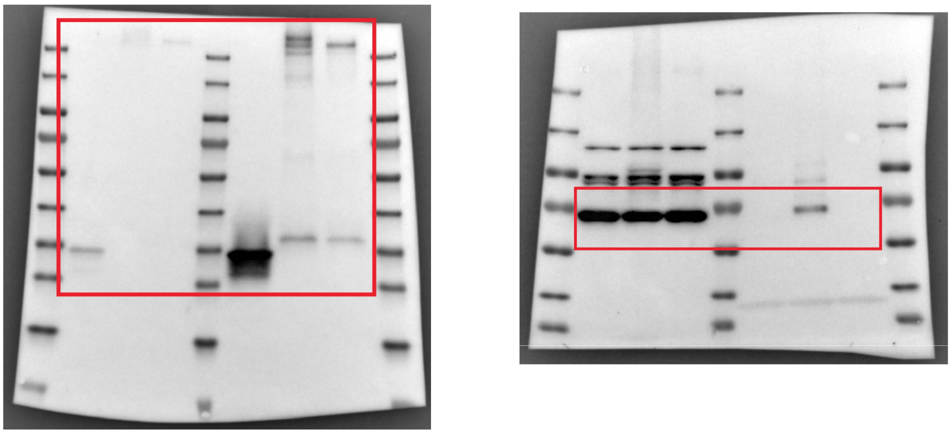

Fig.1.e (left panel)

IP: Flag
WB: Flag

IP: Flag
WB: HA

WB: HA

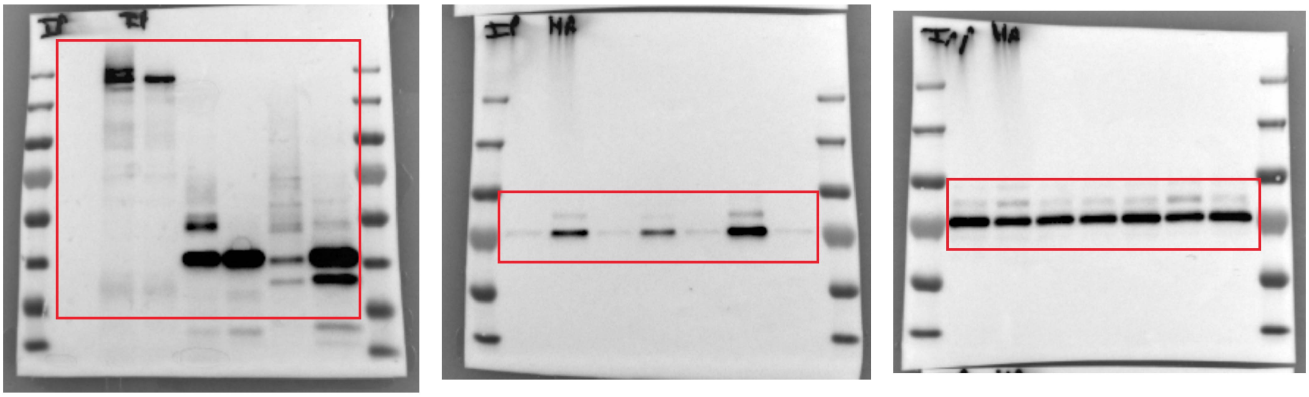

Fig.1.e (right panel)

IP: Flag
WB: Flag

IP: Flag
WB: HA

WB: HA

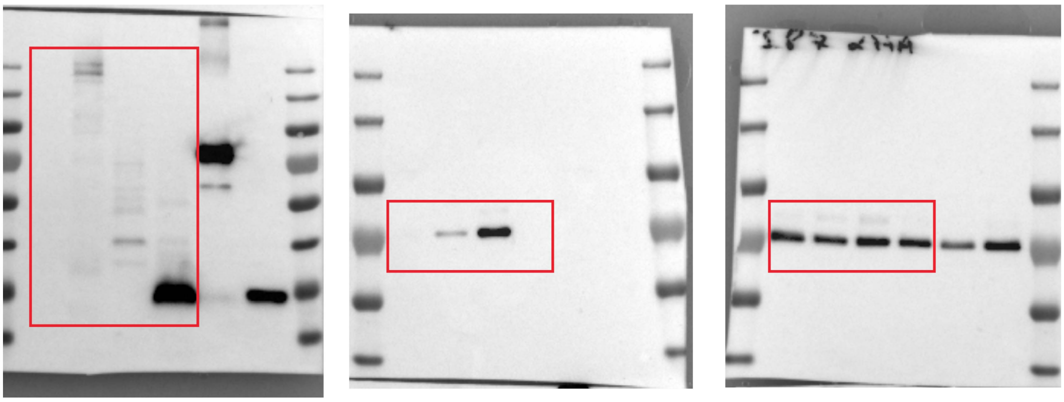

Fig.1.f

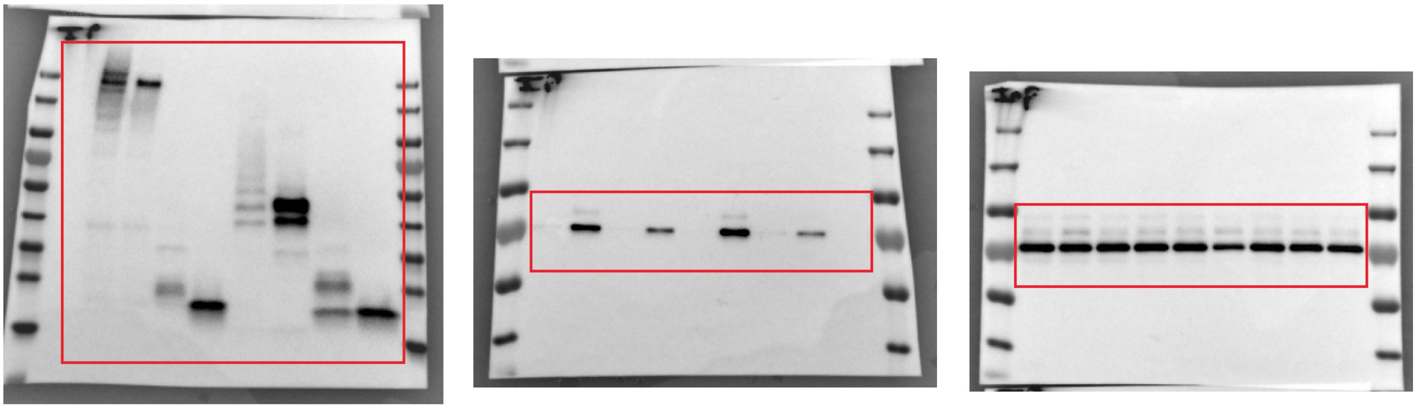

Fig 2.b

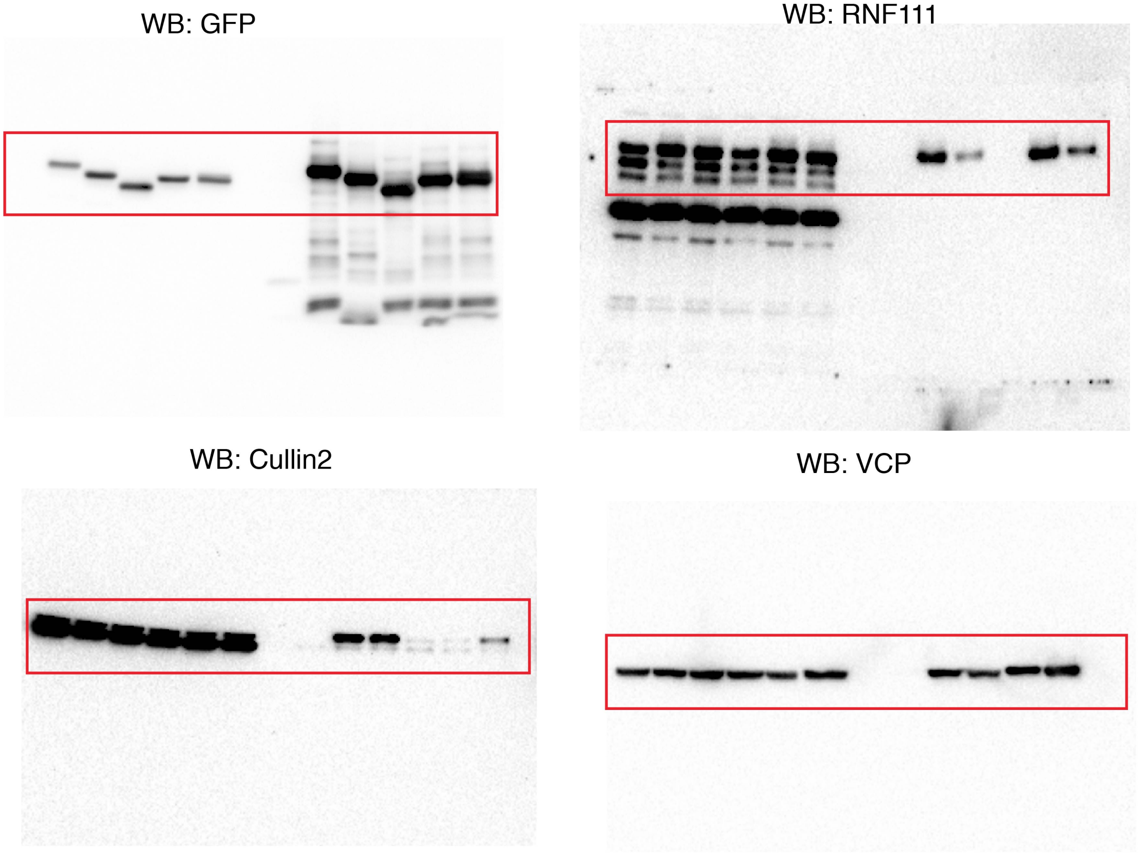

Fig 2.c

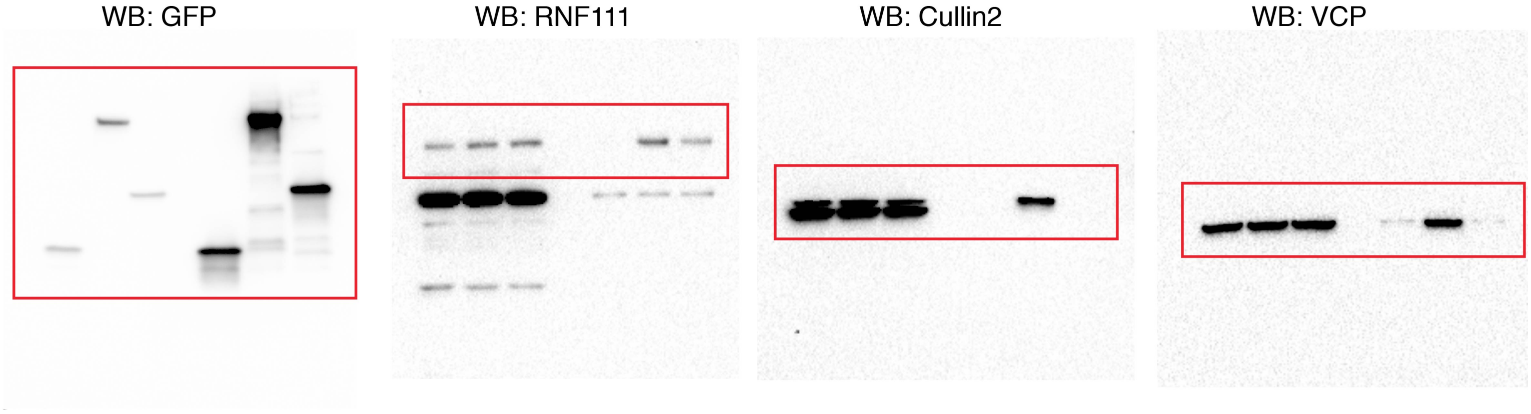

Fig 2.d

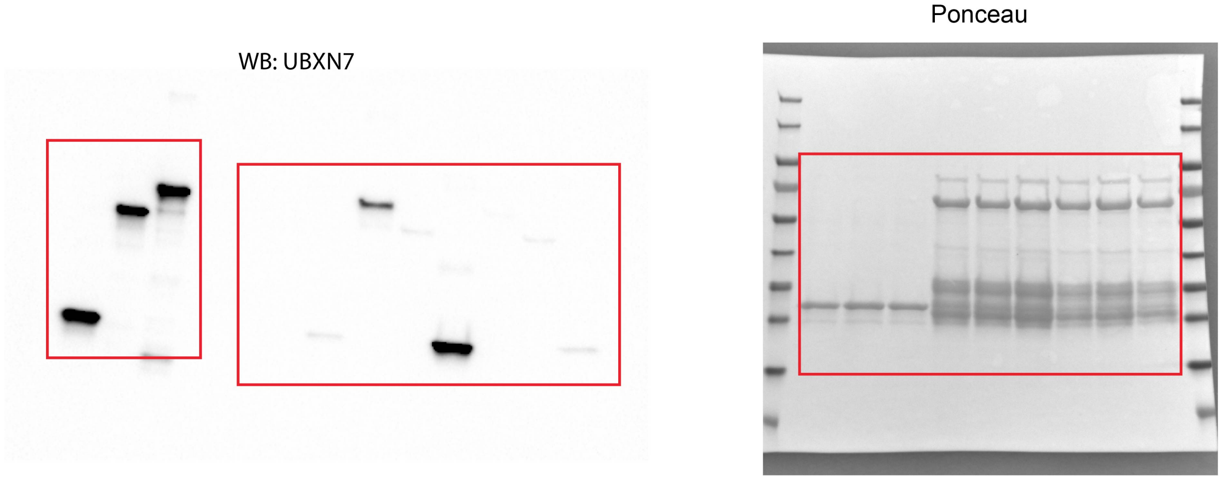

Fig 2.f

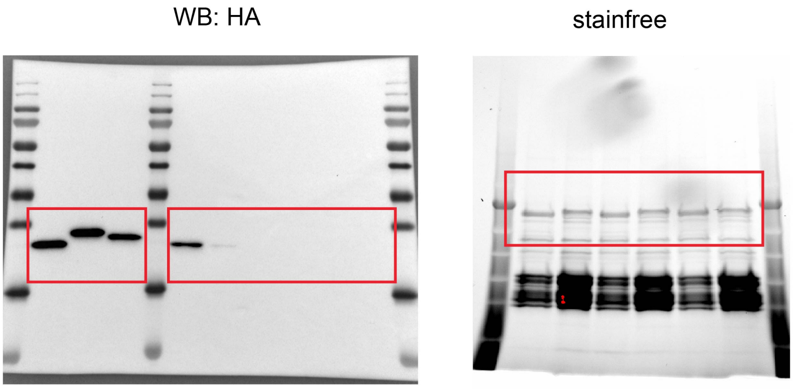

Fig 3.a

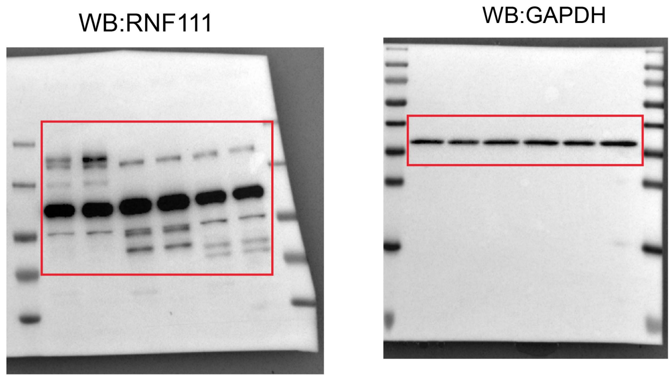

Fig 3.b

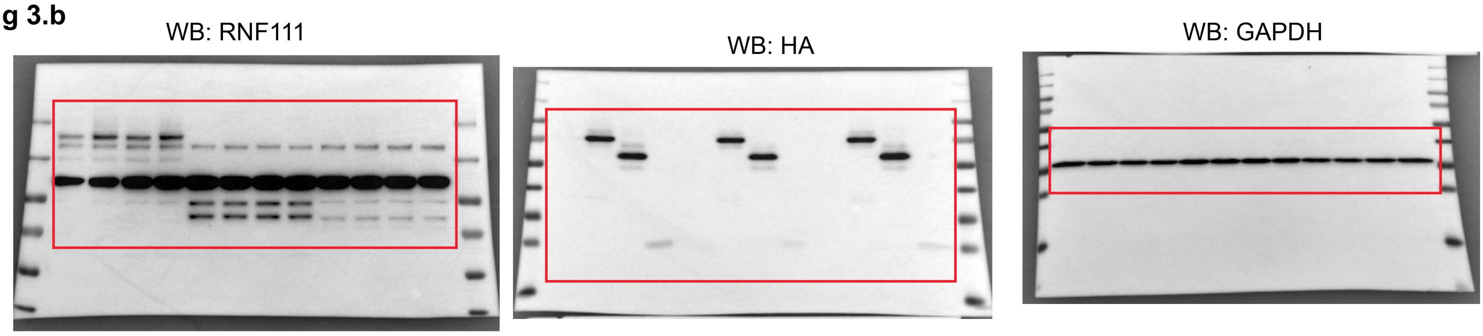

Fig 3.c

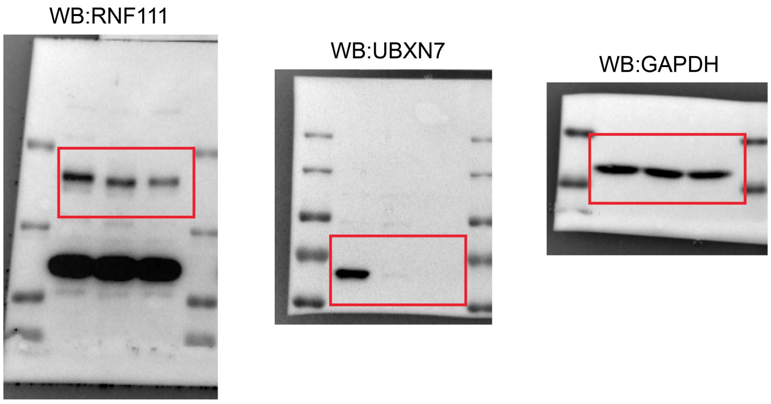

Fig 3.d

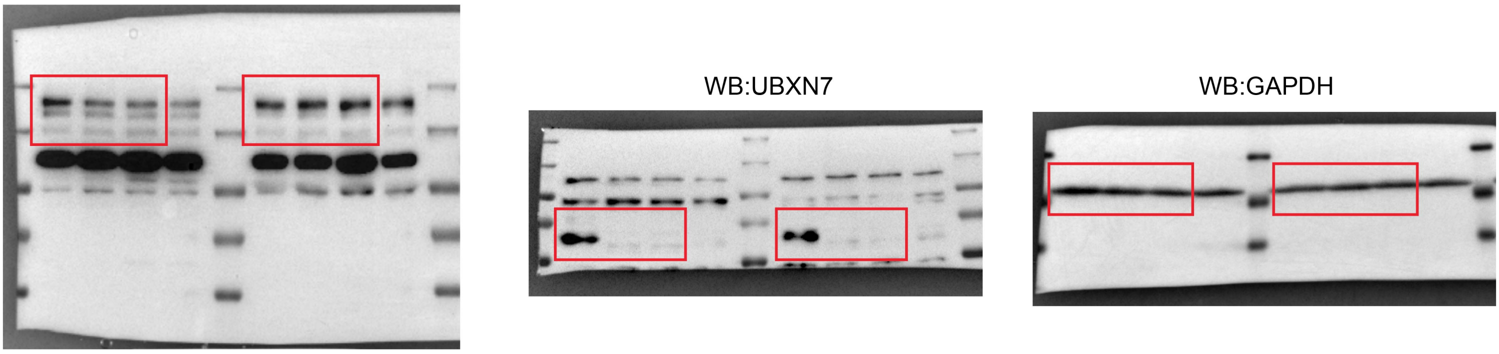

Fig 3.e

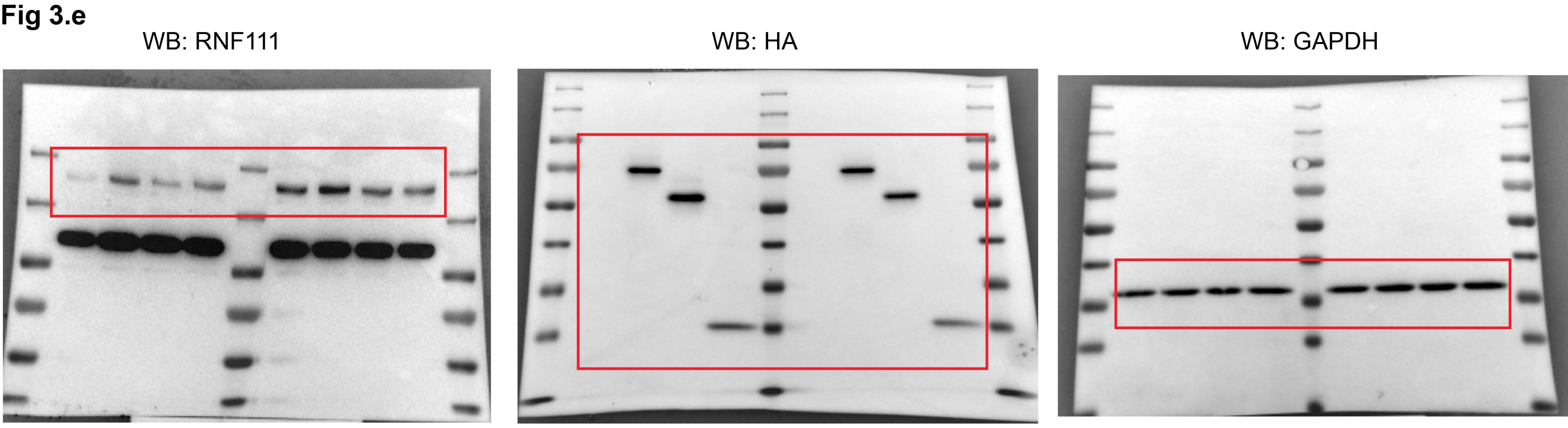

Fig 4. a

WB : Flag

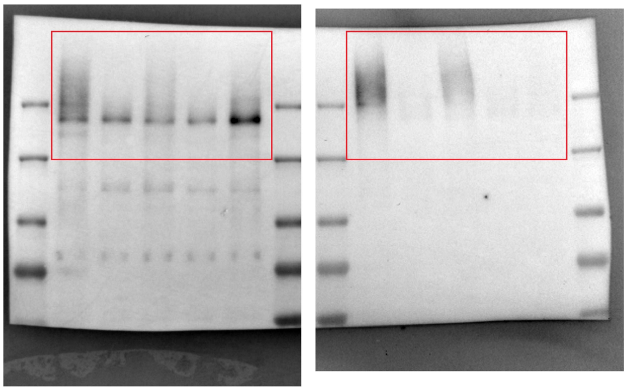

WB : UB

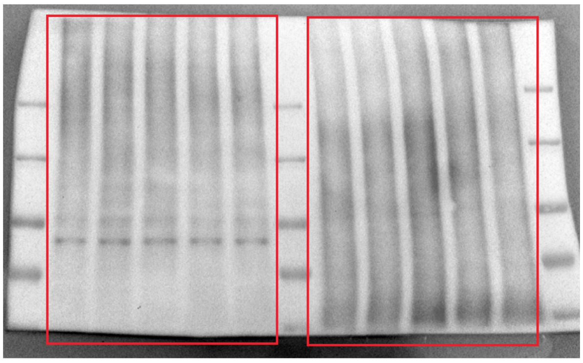

WB : HA

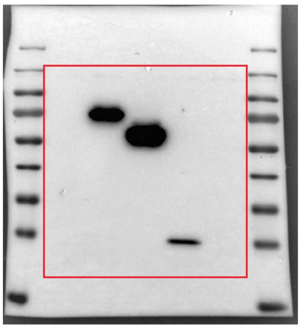

Fig 4. b

WB : RNF111 (Cter)

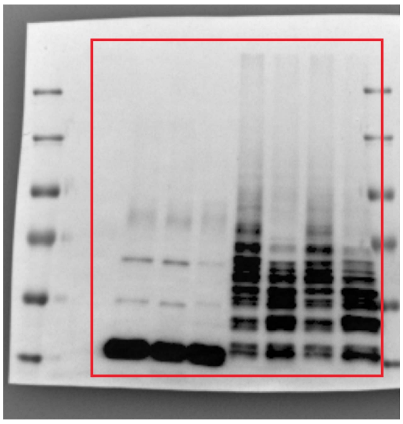

Fig 4. c

WB: UB

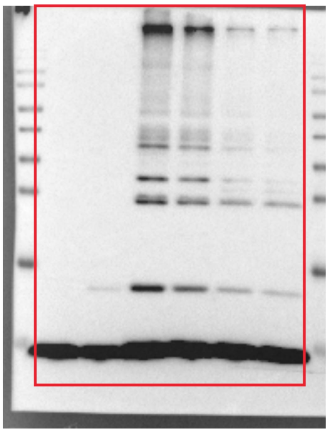

Fig5.a

GST-PD-UB +unbound (upper panel)

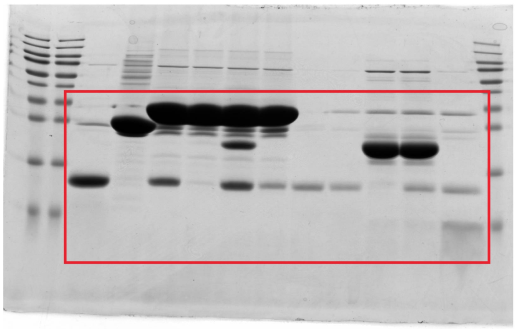

GST-PD-UBE2D2 +unbound (lower panel)

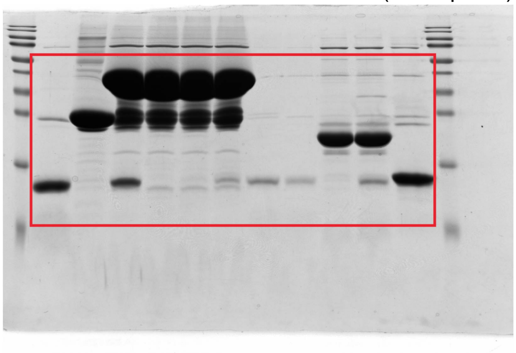

Fig5.b

GST-PD-UBE2D2+unbound

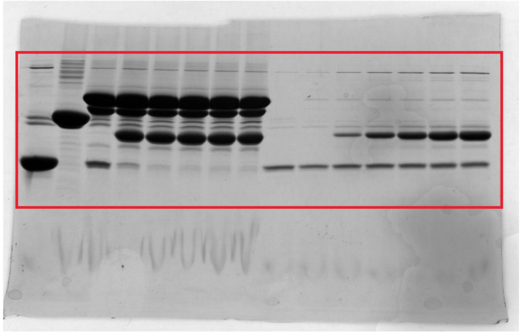

Fig5.c

GST-PD-UBE2 (GST-PD)

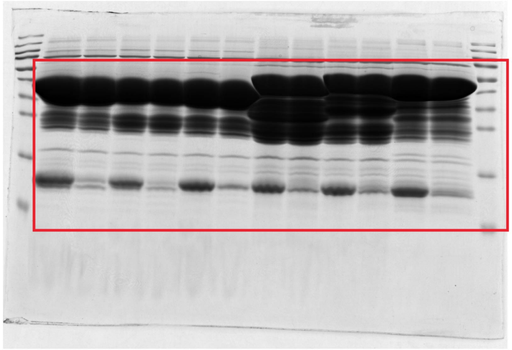

GST-PD-UBE2 (unbound)

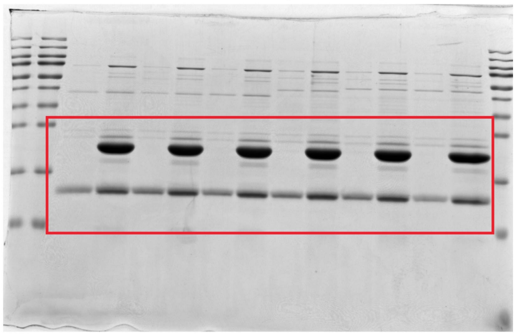

GST-PD-UBE2 (GST-PD + unbound)

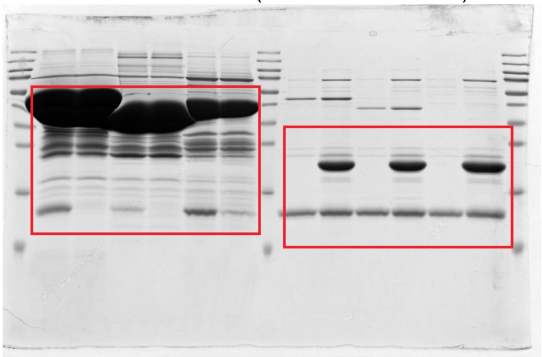

Fig 6.a

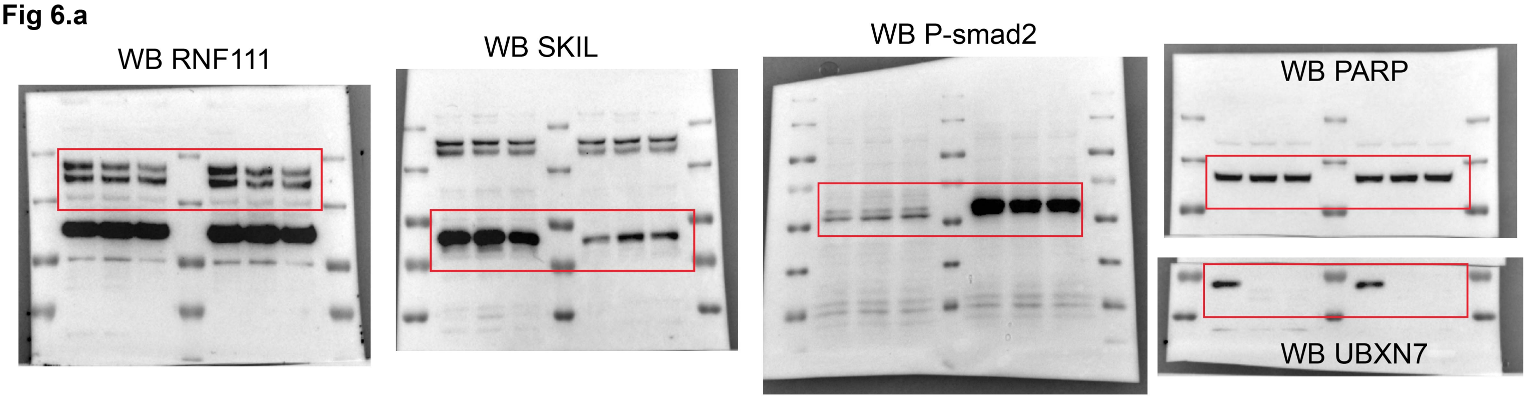

Fig 6.c

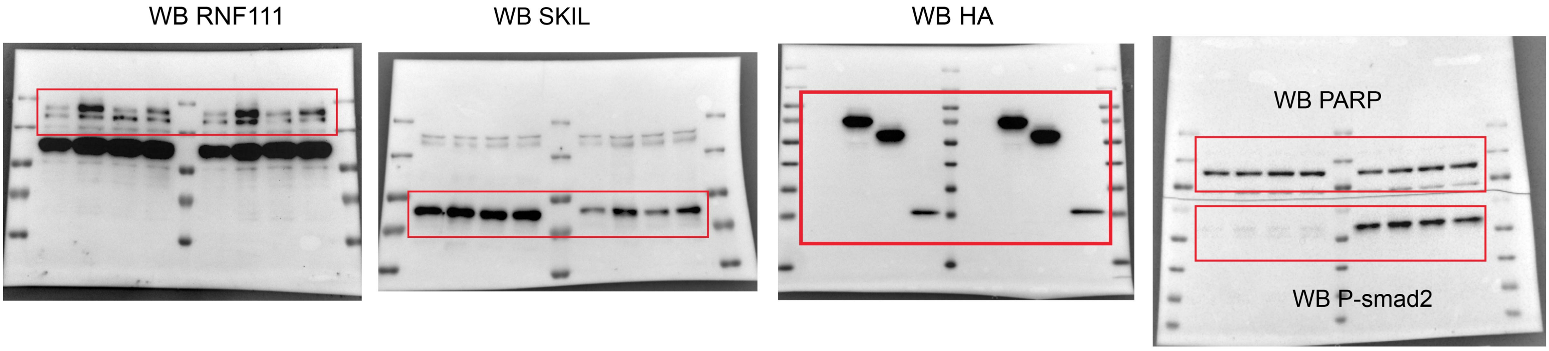

Fig 6.e

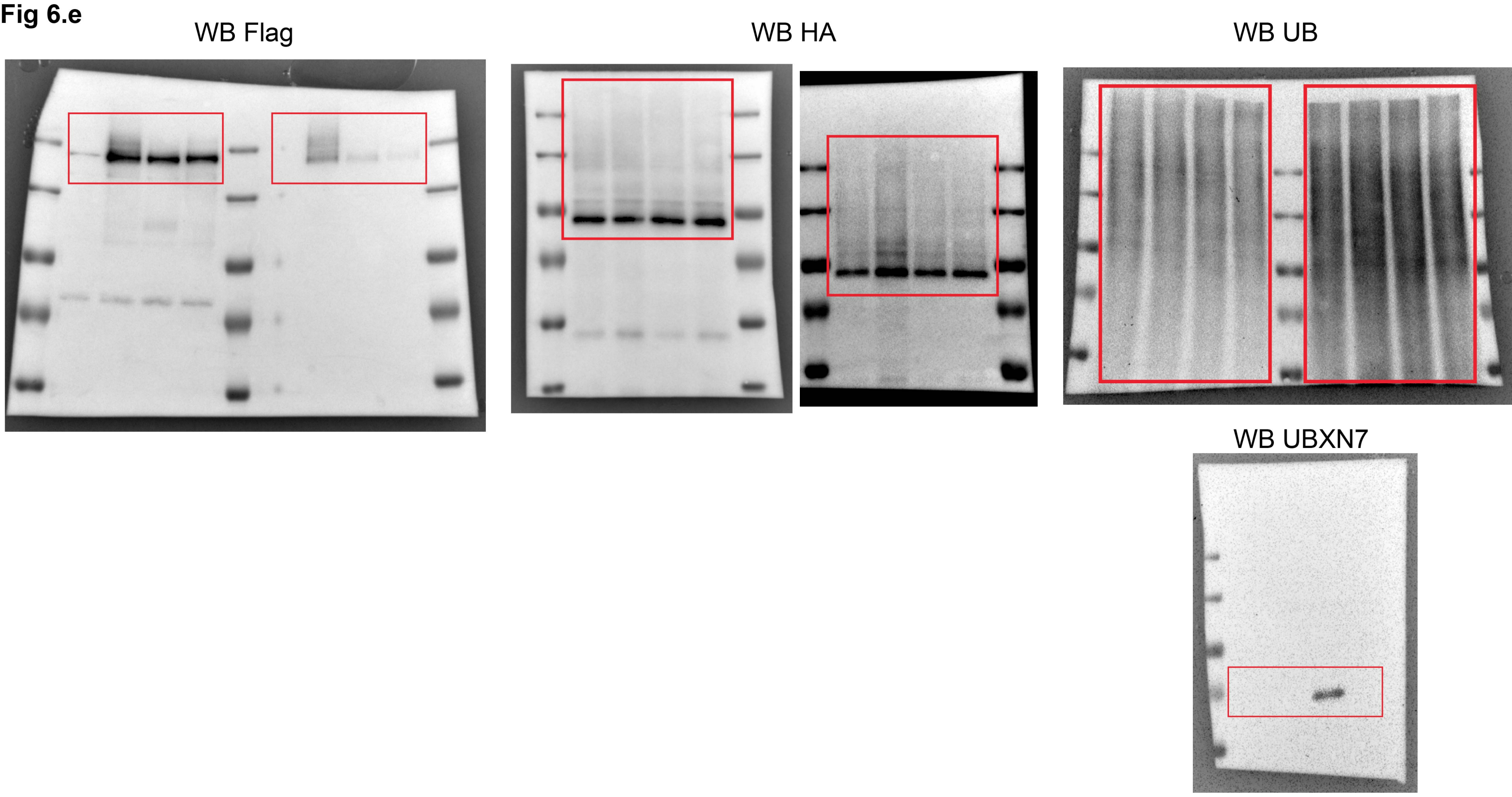

Fig 7.b

WB: RNF111

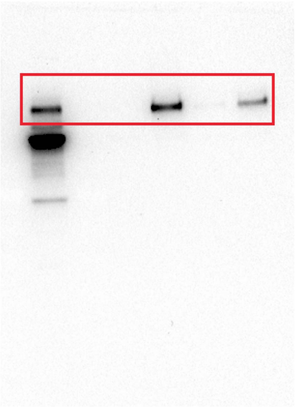

WB: Topors

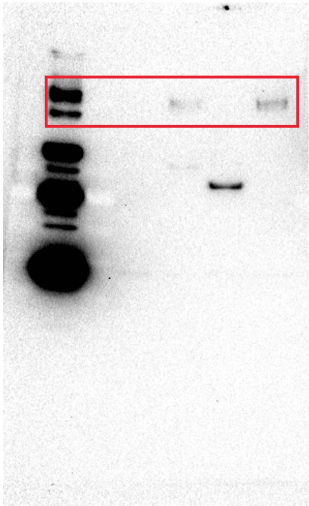

Stain-free

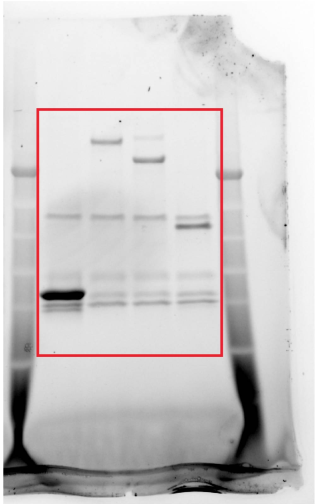

Fig 7.c

WB: Flag

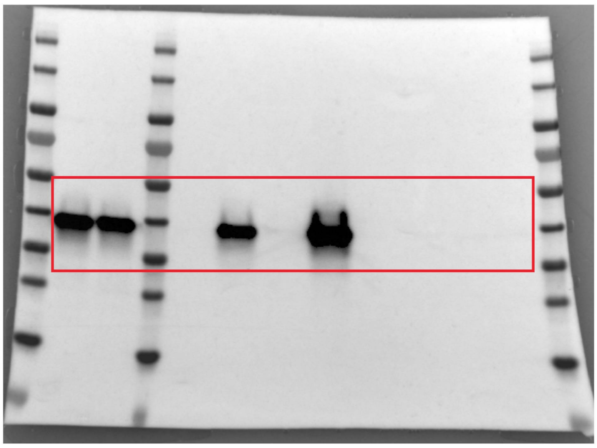

Stain-free

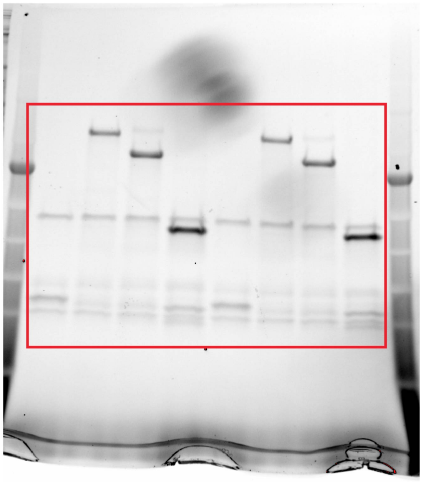

Fig 7.d

Coomassie

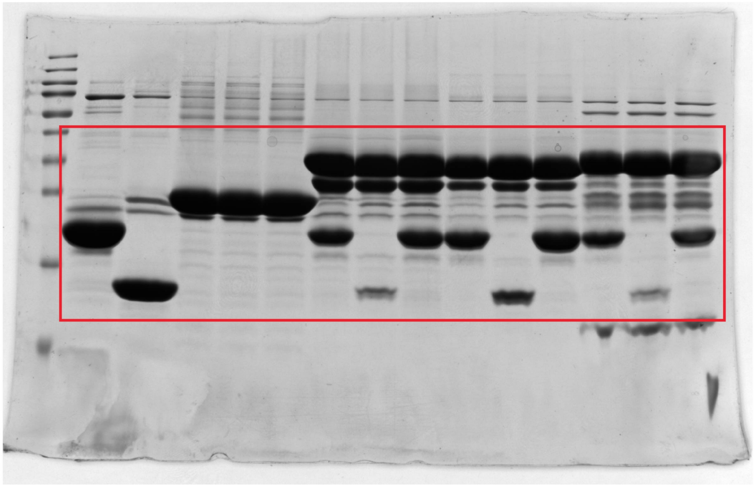

Fig7.e

WB: UB

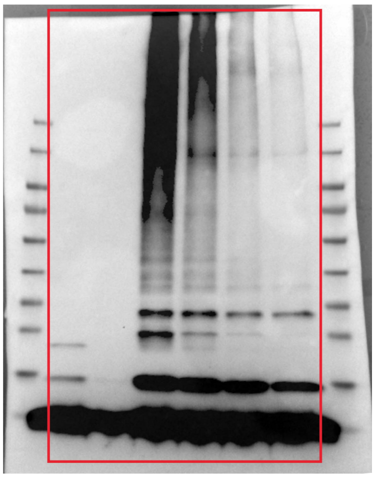

Fig S1.a

IP: Flag
WB: Flag

IP: Flag
WB: HA

WB: HA

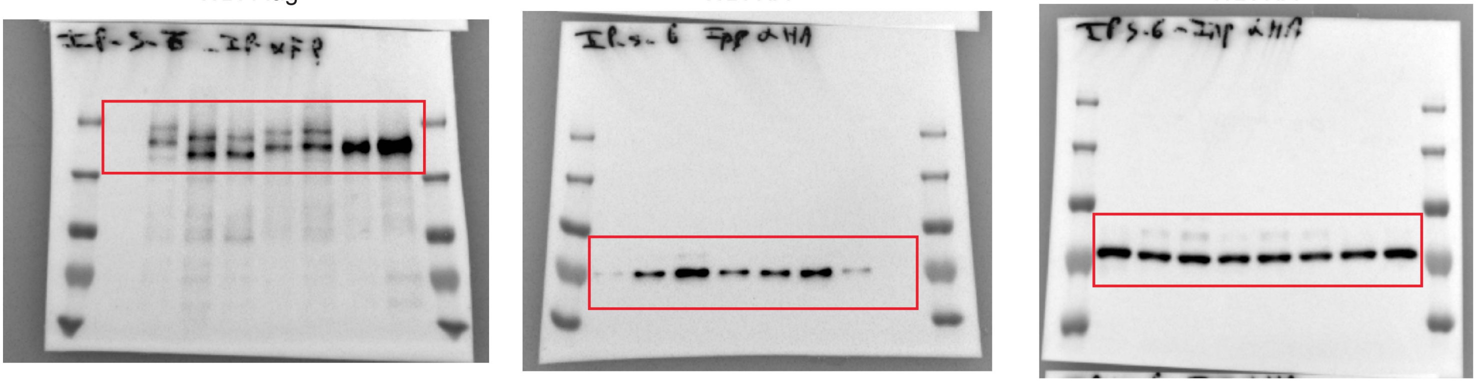

Fig S1.c

IP: HA
WB: Flag

IP: HA
WB: HA

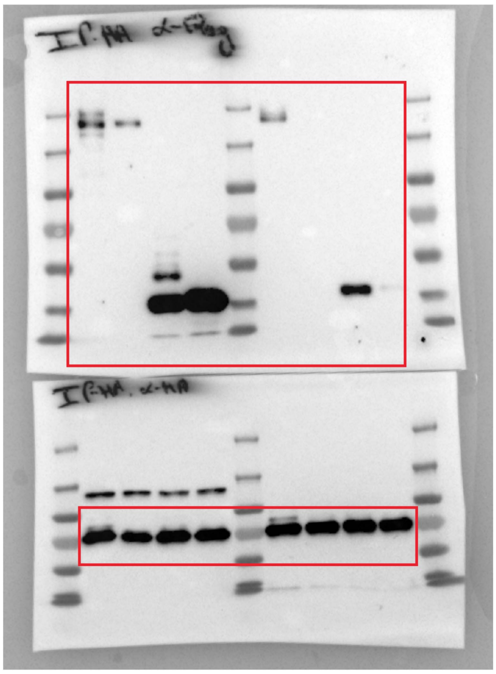

Fig S2.a

Input
WB: HA

GST-PD
WB: HA

GST-PD
stainfree

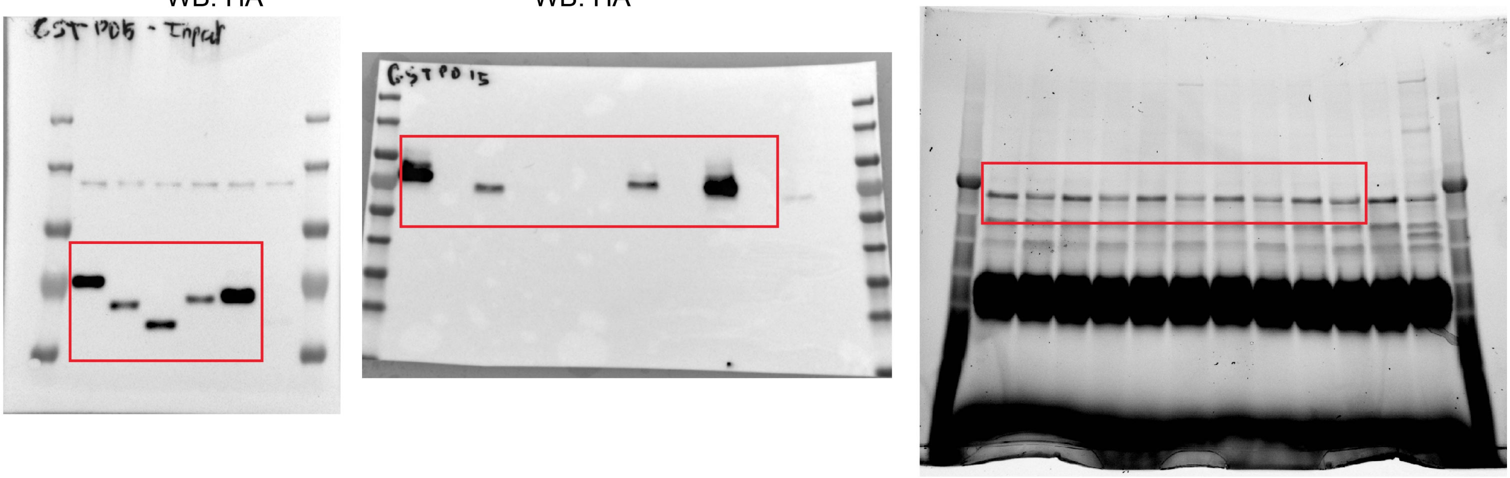

Fig S2.b

Coomassie

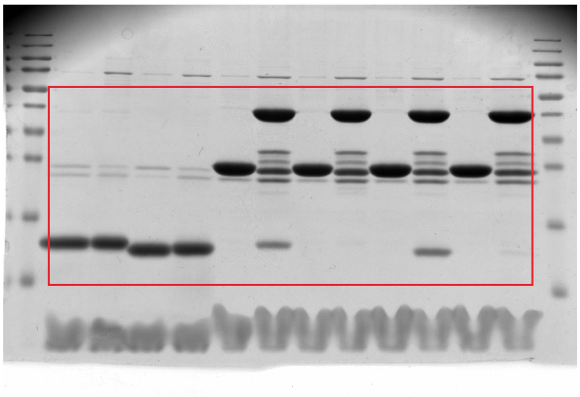

Fig S3.b

Input
WB: HA

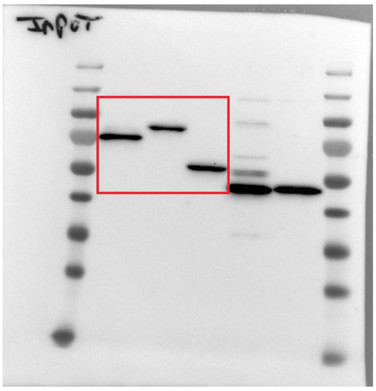

GST-PD
WB: HA

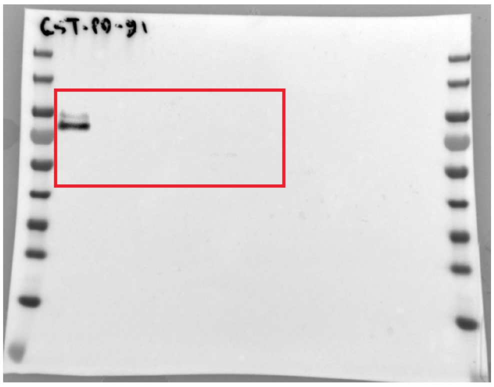

GST-PD
stainfree

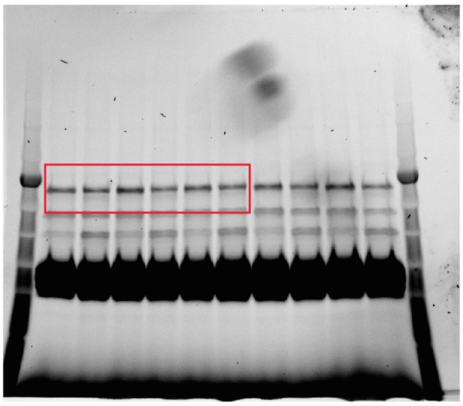

Fig S6

Coomassie

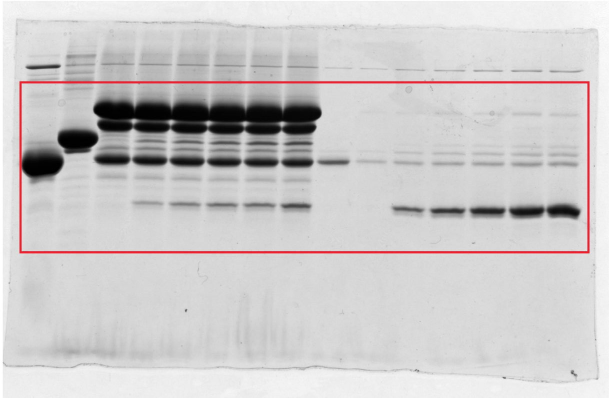

Fig S7

WB : HA

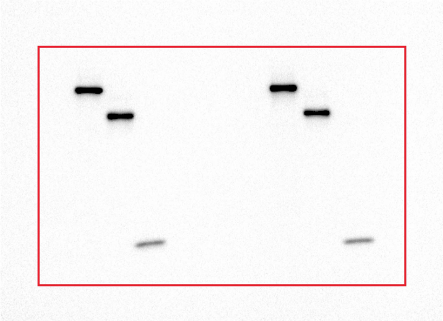

WB : PARP

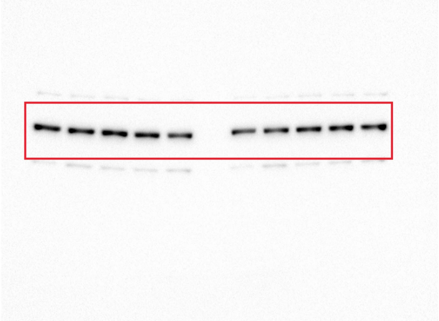

WB : P-Smad2

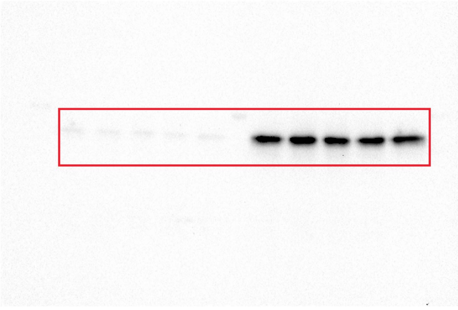

WB : SKIL

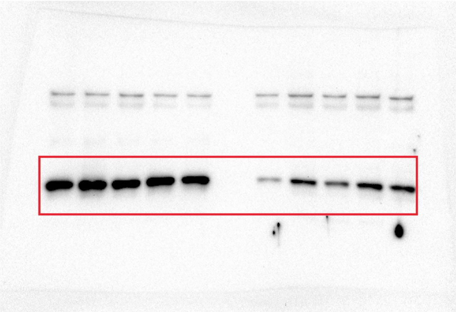

WB : RNF111

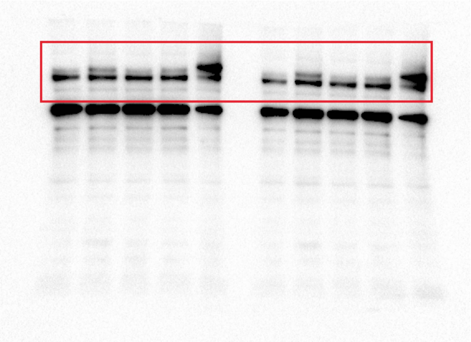

Fig S8

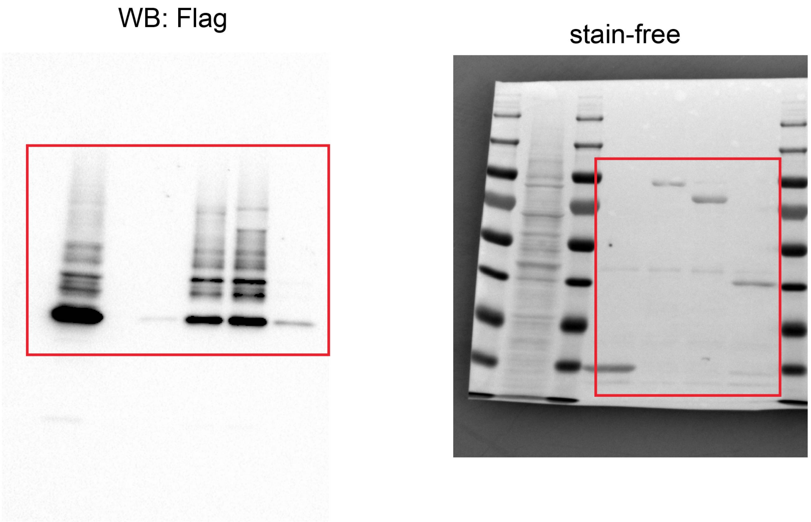

Fig S9.b

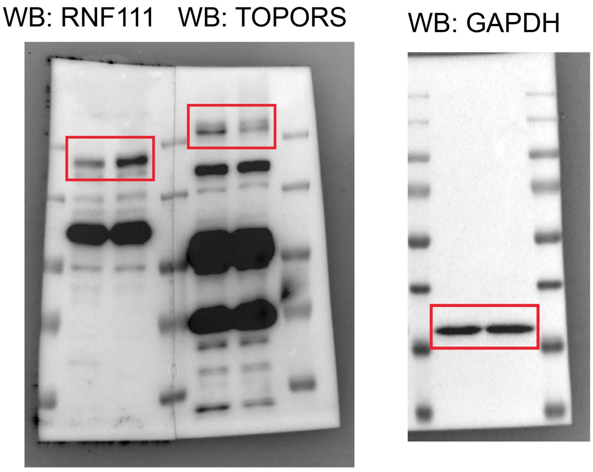

Fig S9.c

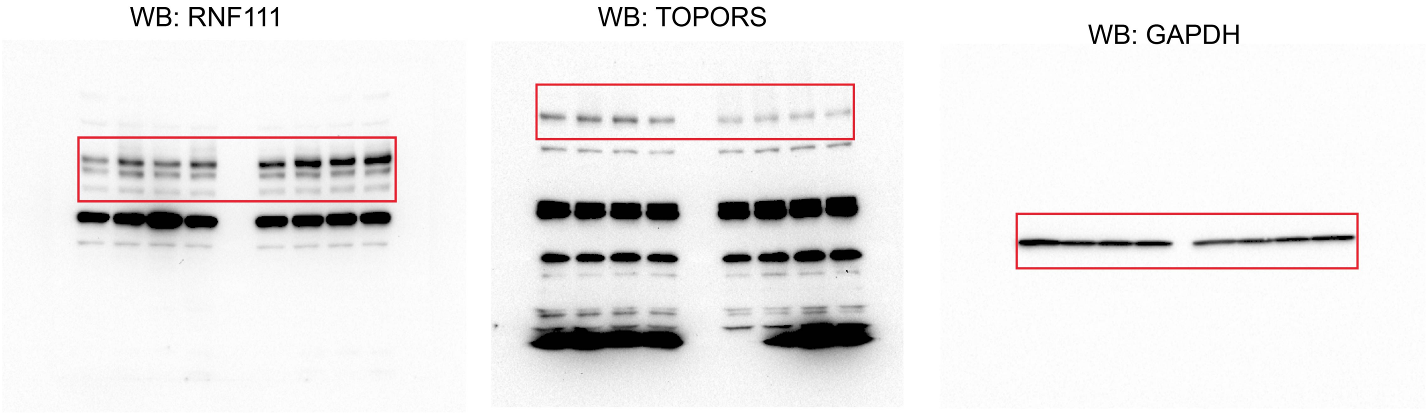

Supplement: Supplementary file 12 — Additional file 12. Figure S10. Images of the original blots presented in this study. [file 12915_2023_1576_MOESM12_ESM.pdf]
